# Supplementary material for: Topological barrier to Cas12a activation by circular DNA nanostructures facilitates autocatalysis and transforms DNA/RNA sensing
Source: Nat Commun. 2024 Mar 5;15:1818. doi: 10.1038/s41467-024-46001-8 (PMC10914725; doi:10.1038/s41467-024-46001-8)
Supplement: Supplementary file 1 — Supplementary Information [file 41467_2024_46001_MOESM1_ESM.pdf]

Topological barrier to Cas12a activation by circular DNA nanostructures facilitates autocatalysis and transforms DNA/RNA sensing

Supplementary Information

Fei Deng [a.b,#], Yi Li [a.b,#,*], Biyao Yang [a.b], Rui Sang[a.b], Wei Deng[c], Maya Kansara [d,e,f], Frank Lin [d,g], Subotheni Thavaneswaran [d,e,g], David M Thomas [d,e,f], Ewa M. Goldys [a.b]

[a] Graduate School of Biomedical Engineering, Faculty of Engineering, University of New South Wales, Sydney 2052, Australia
[b] ARC Centre of Excellence for Nanoscale Biophotonics, University of New South Wales, Sydney 2052, Australia
[c] School of Biomedical Engineering, University of Technology Sydney, Sydney, NSW, 2007, Australia
[d] Garvan Institute of Medical Research, Darlinghurst, NSW, Sydney 2011, Australia,
[e] St Vincent's Clinical School, University of New South Wales, Sydney 2011, Australia,
[f] Omico, Australian Genomic Cancer Medicine Centre, University of New South Wales, Sydney 2052, Australia
[g] NHMRC Clinical Trials Centre, University of Sydney, Sydney, NSW, Australia

[#] These authors contributed equally to this work.
* Correspondence: yi.li6@unsw.edu.au;

Supplementary Figures

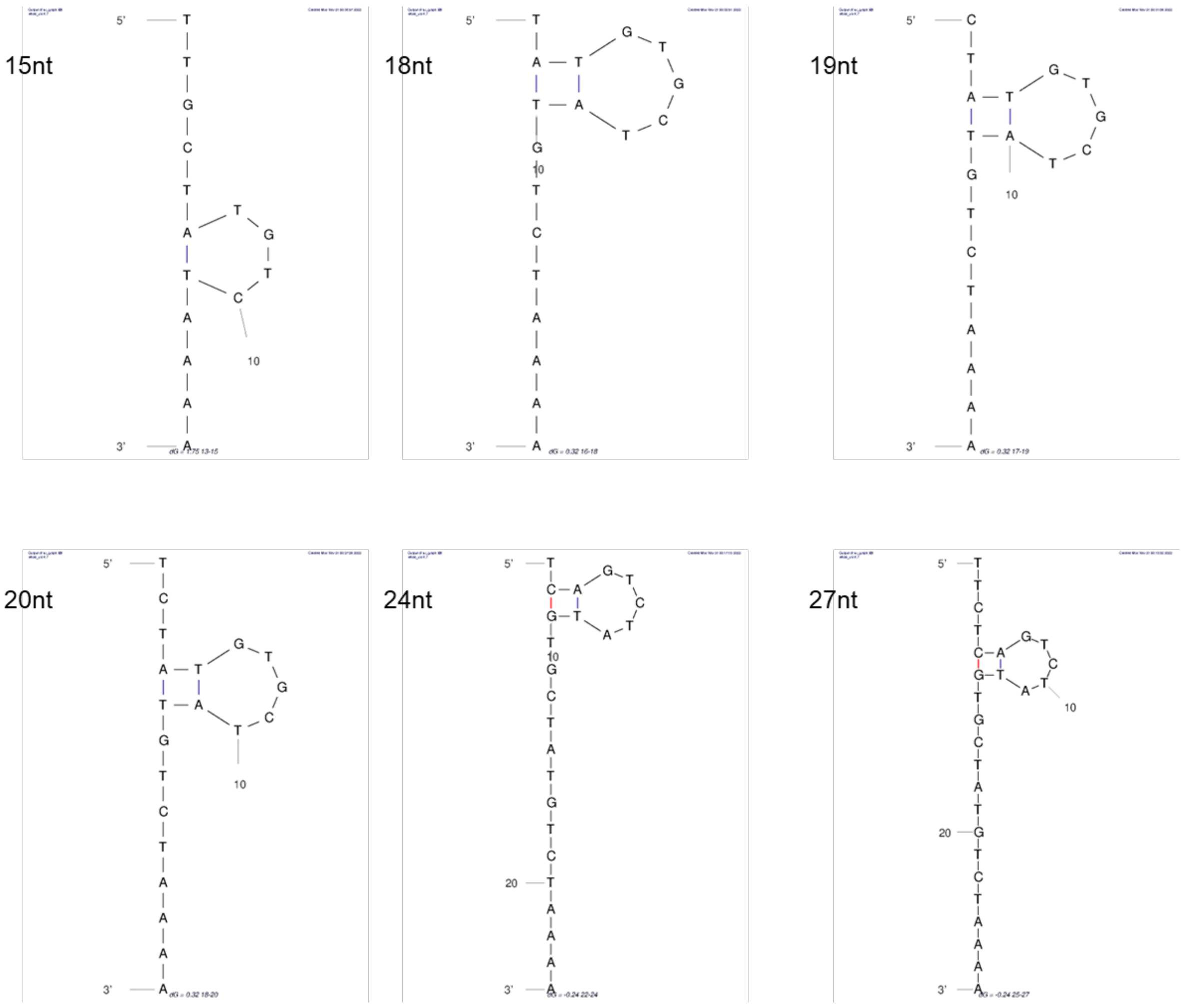

Supplementary Fig. 1 UNAFold simulations show that no complex secondary structures are formed from the triggering ssDNA of different lengths [1,2]. Simulation conditions: 25°C, 50mM NaCl, 10mM $MgCl_2$, corresponding to the 1X NEBuffer 2.1 reaction conditions.

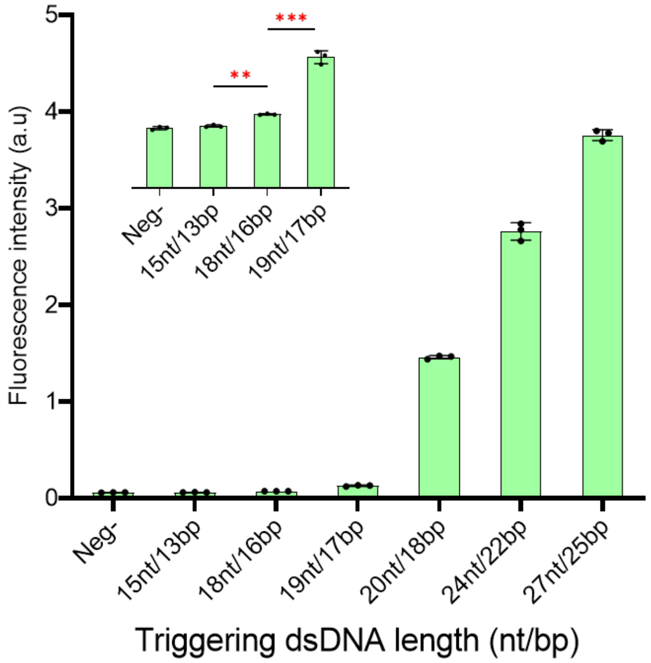

Supplementary Fig. 2 Activation efficiency of Cas12a triggered by L-dsDNA oligos of different lengths. The structures are labelled with their overall ssDNA length/length of dsDNA region. Similarly to the corresponding L-ssDNA oligos, with the overall ssDNA length decreasing from 27 nt to 15 nt (the dsDNA region length decreasing from 25 bp to 13 bp), the activation efficiency of Cas12a *trans*-cleavage decreased as well. Experiment revealed that the L-dsDNA oligo of 19 nt length induced only ~8.8% of *trans*-cleavage activity compared to 20 nt oligo L-dsDNA. There was no significant difference of the fluorescence signals for oligos shorter than 18 nt compared with negative controls without trigger L-dsDNA (Method 2). Error bars represent mean ± SD, where n = 3 independent reactions, * P<0.05, ** P<0.005, *** P<0.001.

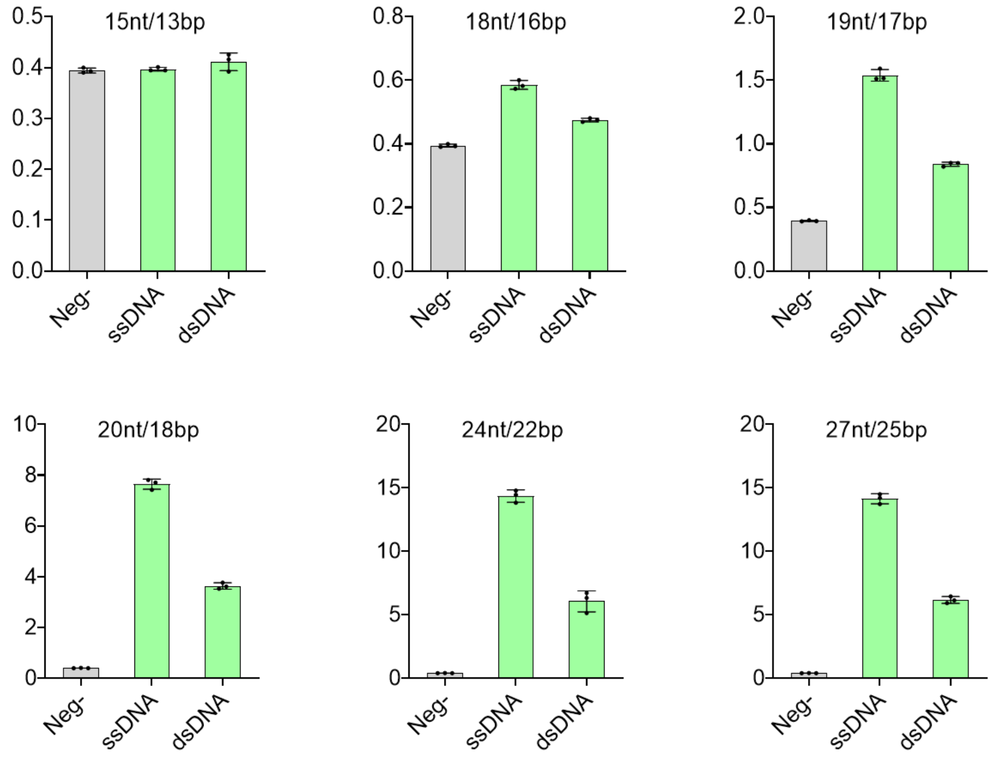

Supplementary Fig. 3 Comparison of Cas12a activation efficiencies between ssDNA and dsDNA (labelled as: length of overall ssDNA/length of dsDNA region). When examining the activation efficiency for the sequence used in this study, we found that, only trigger DNA lengths > 19nt/17bp have been able to increase the fluorescence signal more than by a factor of 3 for both ssDNA and dsDNA triggers (Method 2). Error bars represent mean ± SD, where n = 3 independent reactions.

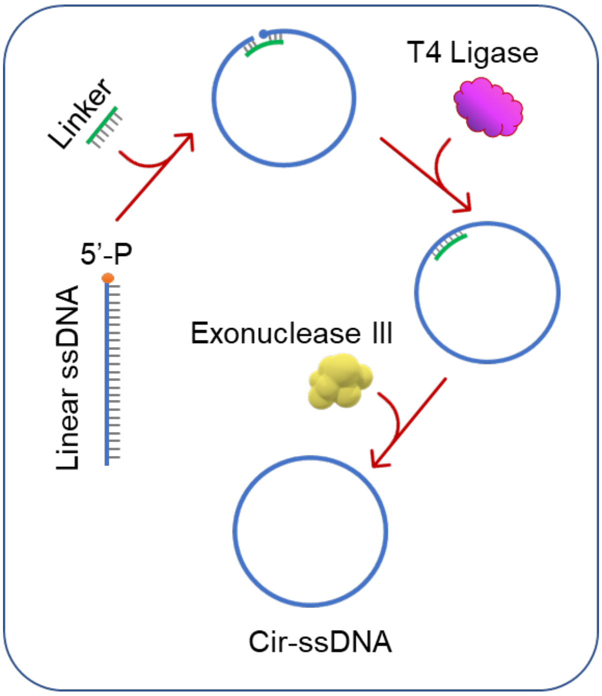

Supplementary Fig. 4 The scheme for T4 DNA ligase-based synthesis of circular ssDNA (Cir-ssDNA). Cir-ssDNA was synthesized in a three-step protocol. Firstly, a 5'-P linear ssDNA oligo with a complementary sequence to the guide RNA of Cas12a RNP (or the target strand), was pulled into a circular shape with the help of a short ssDNA linker, which had complementary sequences to both ends of the linear ssDNA. Then, the break locus was ligated by T4 ligase to form a circular ssDNA structure. Exonuclease III was then applied to remove excess linear ssDNA and linker oligos in the reaction system. (Method 1)

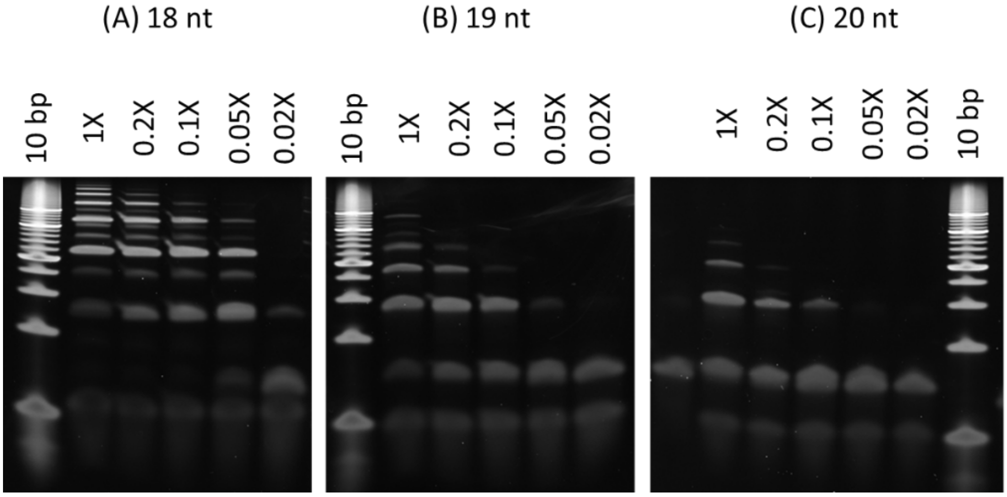

Supplementary Fig. 5 Urea-PAGE electrophoresis verifies the formation of Cir-ssDNA for different lengths of ssDNA oligos (Methods 3, S1). (A) 18 nt 5'-P ssDNA oligo for Cir-ssDNA synthesis. (B) 19 nt 5'-P ssDNA oligo for Cir-ssDNA synthesis. (C) 20 nt 5'-P ssDNA oligo for Cir-ssDNA synthesis. 10 bp = 10 bp DNA ladder, 1X = 1X T4 ligation buffer, 0.2X = 5 times diluted 1X T4 ligation buffer, 0.1X = 10 times diluted 1X T4 ligation buffer, 0.05X = 20 times diluted 1X T4 ligation buffer, 0.02X = 50 times diluted 1X T4 ligation buffer. In agreement with a previous study [3], our data show that, for different length of ssDNA oligos, increasing the dilution times of the 1X T4 ligation buffer during synthesis helps to increase the amount of formed circular ssDNA. However, dilution times higher than 20 times lead to a decrease of the overall efficiency of circular ssDNA synthesis. Hence, buffer dilution by a factor of 5-10 is optimal for our T4 ligase-based Cir-ssDNA synthesis method.

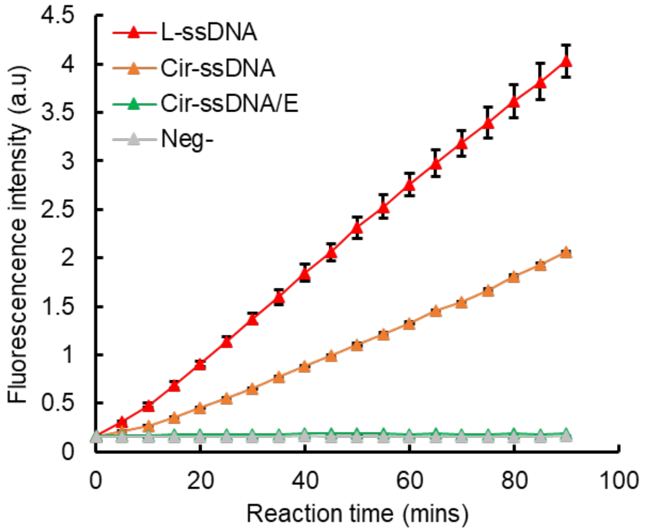

Supplementary Fig. 6 Verification of Cas12a *trans*-cleavage activation by synthesized Cir-mediators. The synthesized Cir-ssDNA structures, without (Cir-ssDNA) or after (Cir-ssDNA/E) exonuclease III treatment, were used as the trigger to activate the Cas12a *trans*-cleavage. Although the Cir-ssDNA without exonuclease III treatment exhibited lower level of Cas12a activation compared to L-ssDNA, the continuously increasing fluorescence signal in untreated Cir-ssDNA compared to exonuclease III-treated Cir-ssDNA also indicates the potential existence of unligated linear ssDNA. Exonuclease III-treated Cir-ssDNA/E shows a significantly reduced activation efficiency due to the digestion of extraneous L-ssDNA. Hence, exonuclease III treatment is necessary to remove the L-ssDNA residues (Method S2). Error bars represent mean ± SD, for n = 3 independent reactions.

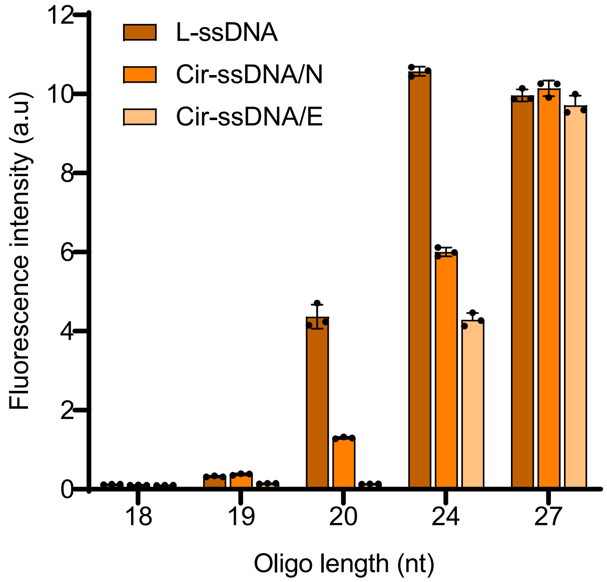

Supplementary Fig. 7 Comparison of Cas12a activation patterns with synthesized Cir-ssDNA of different lengths, with and without exonuclease III treatment. In comparison to the respective L-ssDNA (Dark brown), the activation efficiency of synthesized Cir-ssDNA oligos, including exonuclease III-treated Cir-ssDNA/E (with exonuclease III treatment, light brown) and Cir-ssDNA/N (no exonuclease III treatment, medium brown) show a significantly decreased signal intensity with decreasing oligo length from 27 nt to 20 nt. All types of oligos shorter than 20 nt produce limited Cas12a RNP activation. For 20 nt oligos, the activation of Cas12a RNP by Cir-ssDNA/E was significantly reduced by more than 90% comparing to its linear form. This oligo length produced the highest difference of the Cas12a RNP activation efficiency changes, hence, 20 nt was used for further study. (Method S2) Error bars represent mean ± SD, for n = 3 independent reactions.

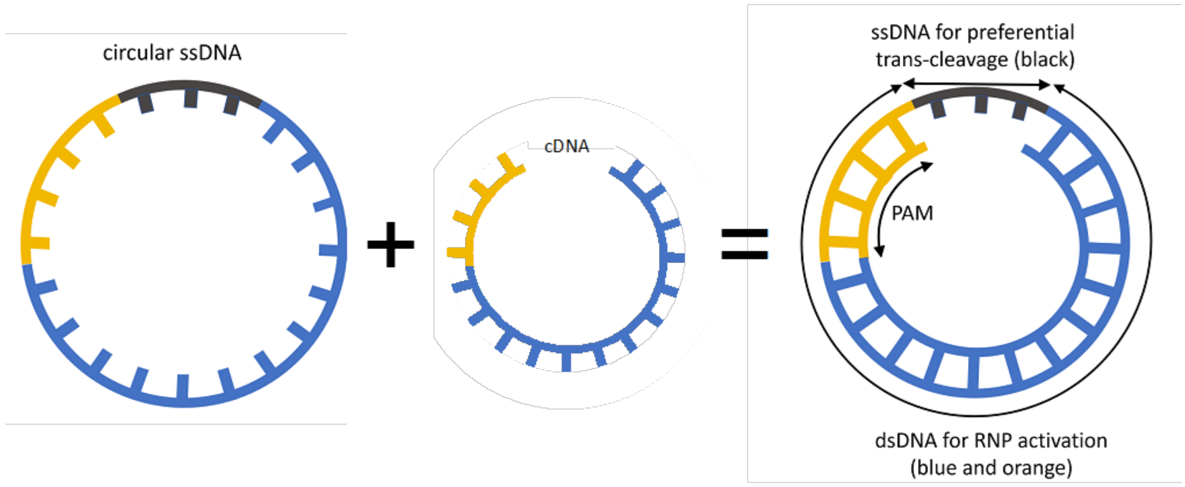

Supplementary Fig. 8 Schematics for combining Cir-ssDNA with cDNA to form the Cir-mediator DNA nanostructure. To form the final Cir-mediator DNA nanostructure, a slightly shorter complementary ssDNA (cDNA) oligo was added to the Cir-ssDNA. The cDNA covers the PAM region (orange) and also the Cas12a RNP2 targeting sequence (blue) to form a dsDNA region on the Cir-mediator. The remaining single strand region (black) allows the activated Cas12a to easily break and open the circle through *trans*-cleavage.

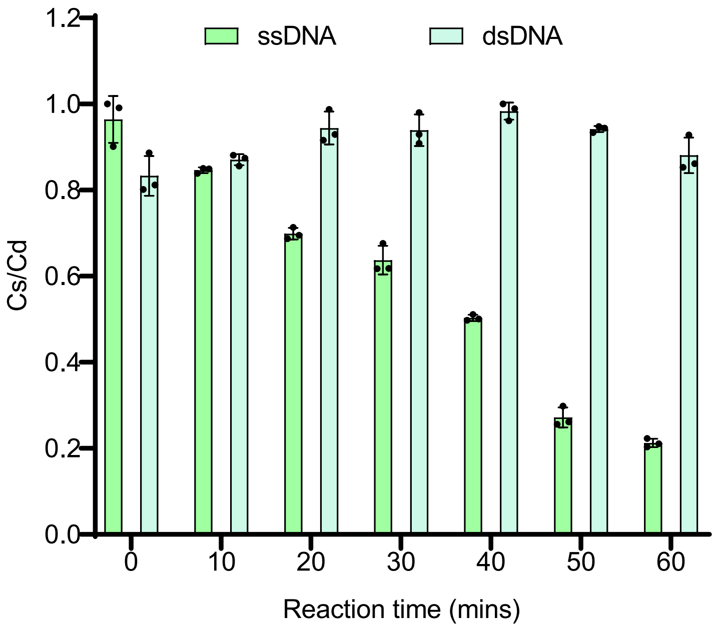

Supplementary Fig. 9 Quantification of band intensity changes in Fig. 2G. Cs = the colour intensity of the band with sequentially increased reaction time, Cd = the colour intensity of the band with the defined start point. This figure is generated from Fig. 2G with image intensity analysis by Image J. Green bar = ssDNA, light blue bar = ssRNA. Error bars represent mean ± SD, for n = 3 independent measurements.

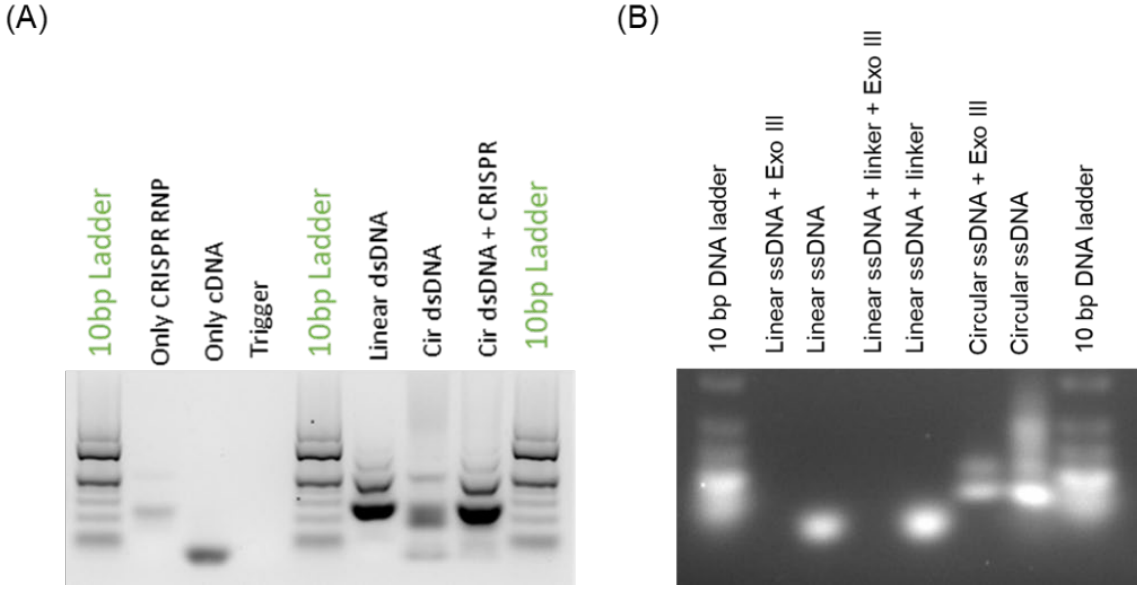

Supplementary Fig. 10 (A) Integrity of the *trans*-cleavage treated Cir-mediator. Electrophoresis gel demonstrates that the Cir-mediator is not completely degraded after treatment with pre-activated Cas12a RNPs, as indicated in the band of "Cir dsDNA + CRISPR". Description of lanes as in the figure, Cir dsDNA = Cir-mediator (Method 12). (B) Treatment of Circular ssDNA with exonuclease III. The linear and circular ssDNA has been exposed to exonuclease III, which degrades ssDNA oligos with a free 3' terminal. The result here indicates that only circular ssDNA can withstand the presence of exonuclease III, which indicates a successful formation of the circular DNA structure – as a circularized ssDNA strand does not have an accessible 3' terminal.

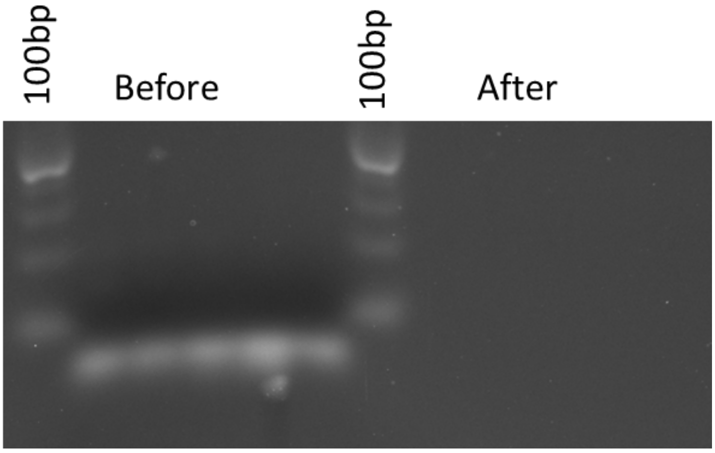

Supplementary Fig. 11 Treatment of Cir-ssDNA with previously activated Cas12a RNP. Five different batches of Cir-ssDNA were synthesized using same synthesis protocol (Method S1), and then they were treated with previously activated Cas12a RNPs for 60 mins at room temperature (Method S3). 100bp = 100 bp DNA marker. The results show no visually identifiable fragments on the gel with molecular weight under the lowest band of 100 bp DNA ladder. This highlights the need to protect the Cir-ssDNA structures from complete degradation by these RNPs which we accomplished by integrating them with c-DNA, hence forming Cir-mediators.

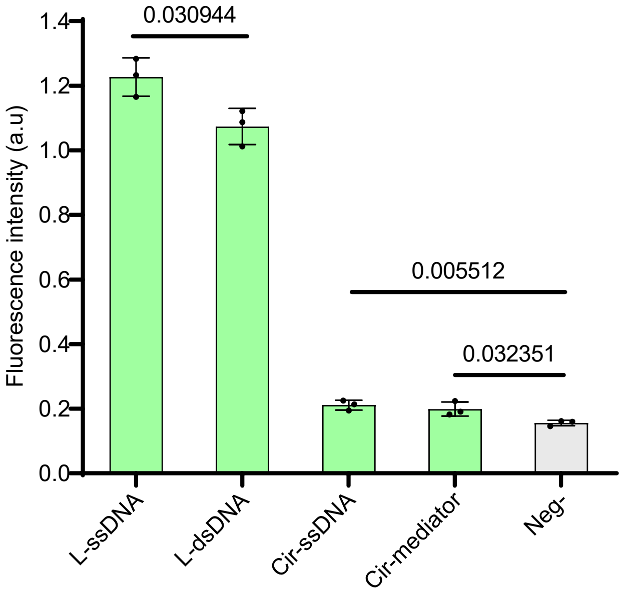

Supplementary Fig. 12 Comparison of Cas12a activation for different DNA molecular structures prepared from 20 nt L-ssDNA. The results show that compared to the linear conformations (L-ssDNA or L-dsDNA), the circular DNA structures (Cir-ssDNA or Cir-mediator) have significantly lower Cas12a *trans*-cleavage activation efficiency. After adding the cDNA to Cir-ssDNA to form the Cir-mediator DNA nanostructures, the restriction of Cas12a activation persists. (Method 2) Error bars represent mean ± SD, for n = 3 independent reactions.

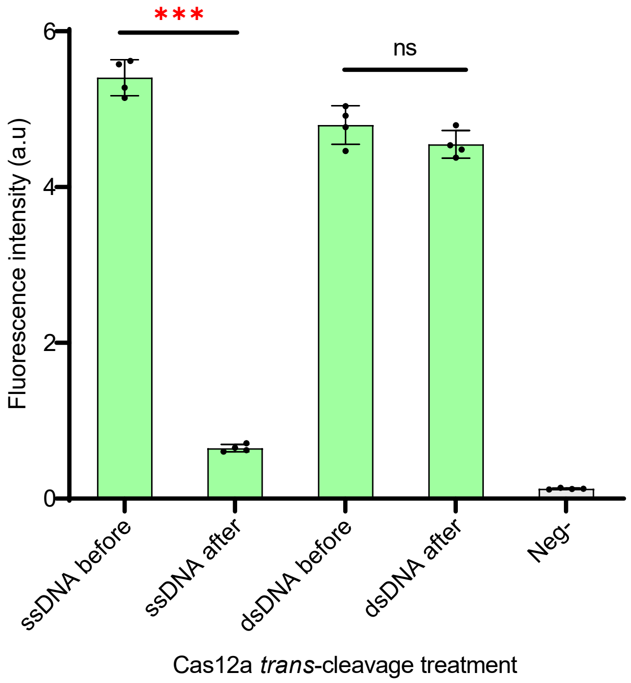

Supplementary Fig. 13 The Cas12a activation efficiency of oligos after treatment with previously activated Cas12aRNPs capable of *trans*-cleavage. Compared to the trigger ssDNA oligos which produce a significantly decreased fluorescence signal intensity after Cas12a *trans*-cleavage treatment, the trigger dsDNA oligos do not produce significant changes of the assay signal in the same conditions. (Method S3) Error bars represent mean ± SD, where n = 3 independent reactions, * P<0.05, ** P<0.005, *** P<0.001.

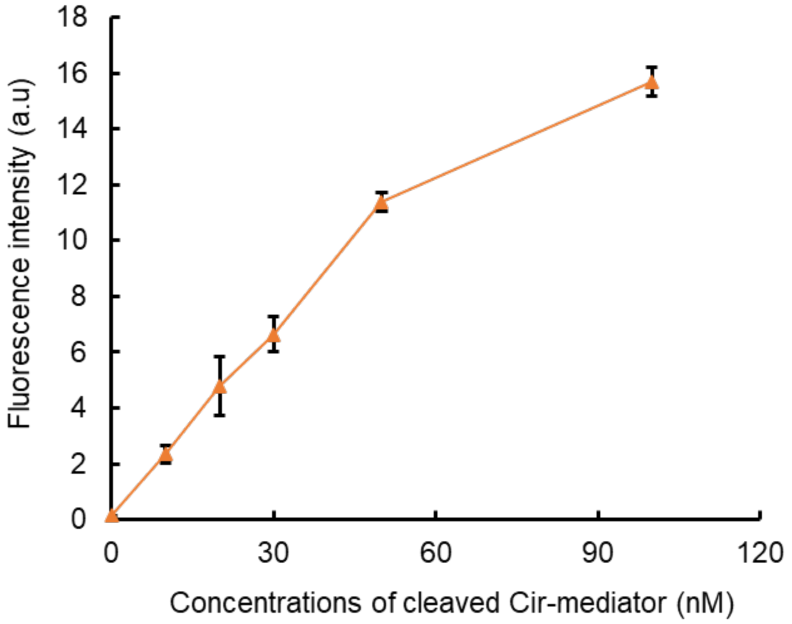

Supplementary Fig. 14 Tests of different concentrations of *trans*-cleavage-treated Cir-mediators for the effectiveness of downstream Cas12a RNP2 activation. Different concentrations of Cir-mediators have been exposed to previously activated Cas12a RNPs for linearization. Subsequent to that, the same amount of cleavage product was used for further Cas12a RNP2 activation. The results indicate that with increasing numbers of linearized Cir-mediators, the downstream Cas12a activation also increases correspondingly. (Method S4) Error bars represent mean ± SD, for n = 3 independent reactions.

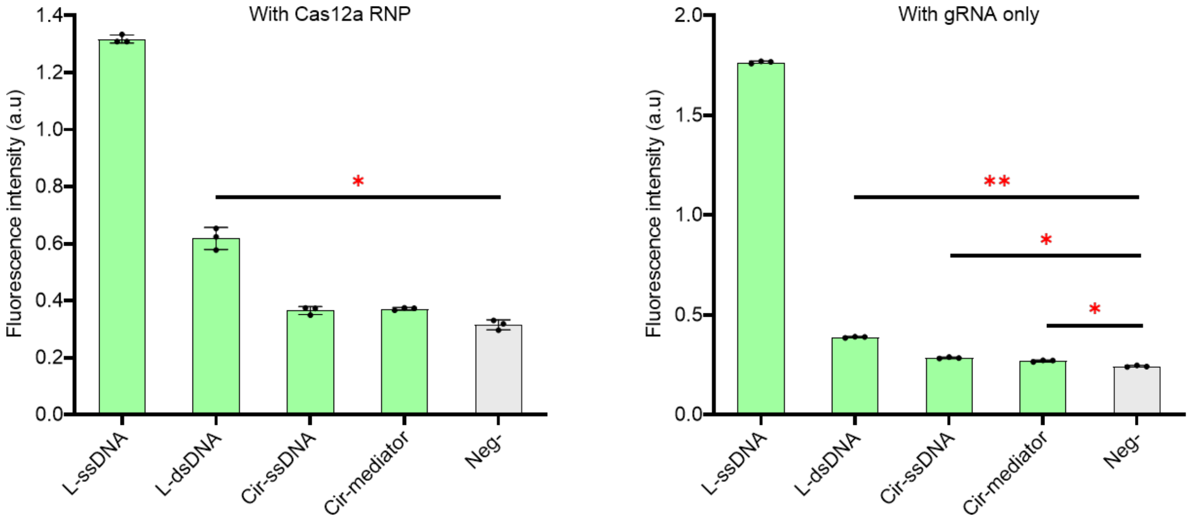

Supplementary Fig. 15 The results of FRET test of the interaction of different DNA molecular structures. The FRET results indicate that for all four different DNA structures (L-ssDNA, L-dsDNA, Cir-ssDNA and Cir-dsDNA) with the sequence targeted by the Cas12a RNP, the interaction to its corresponding Cas12a RNP follow a similar trend, with the affinities of L-ssDNA> L-dsDNA > Cir-ssDNA ≈ Cir-dsDNA, regardless of the presence of Cas12a RNP. (Method 6) Error bars represent mean ± SD, for n = 3 independent reactions, * P<0.05, ** P<0.005, *** P<0.001.

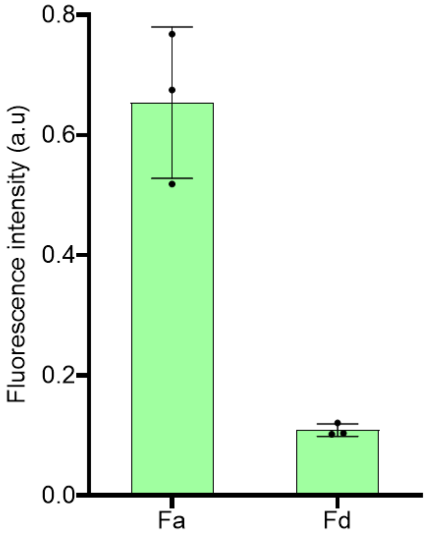

Fa	*Fd*	$E_{FRET} = \frac{Fa}{Fd+Fa}$
653888.33 ± 126058.44	108889.67 ± 10350.18	0.86 ± 0.04

Supplementary Fig. 16 The FRET efficiency for the Cy3-Cy5 pair used in this study. Based on the FRET efficiency equation, $E_{FRET}=Fa/(Fd+Fa)$, where *F*a is the acceptor (Cy5), and *Fd* is the donor (Cy3) emission signal, the measured fluorescence intensities of the Cy3-Cy5 solution at *E*x= 520 nm and *E*m= 560 nm was used as *F*d, and the fluorescence intensities of the Cy3-Cy5 solution at *E*x= 520 nm and *E*m= 666 nm was used as *F*a. The calculated E_{FRET} is 0.86 ± 0.04. (Method S5) Error bars represent mean ± SD, for n = 3 independent reactions.

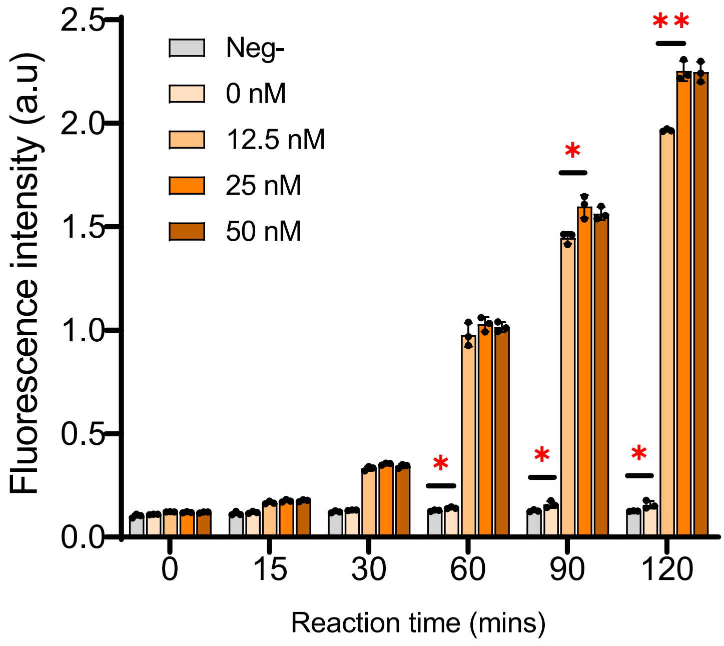

Supplementary Fig. 17 *Trans*-cleavage activity levels of AutoCAR-1 system with different concentrations of Cir-mediators. Compared with the CRISPR/Cas12a reaction without the presence of Cir-mediators (0 nM, light brown), all the AutoCAR-1 reactions with different concentrations of Cir-mediators (12.5 nM, 25 nM, 50 nM) produced increasing fluorescence signal, which indicated elevated reporter *trans*-cleavage activity. “Neg-” (grey bar) represents the reaction mixture without trigger DNA and Cir-mediators, and with the same concentration of reporters. In addition, as shown in the figure, the reporter *trans*-cleavage activity of AutoCAR-1 is also significantly increased with increasing Cir-mediator concentration. (Method S6) Error bars represent mean ± SD, for n = 3 independent reactions, * P<0.05, ** P<0.005, *** P<0.001.

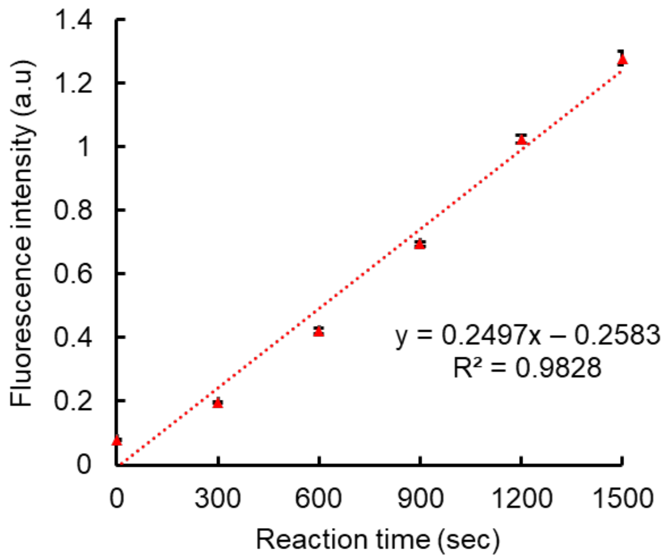

Supplementary Fig. 18 Standard CRISPR/Cas12a *trans*-cleavage reaction pattern. In a standard CRISPR/Cas12a *trans*-cleavage reaction (Method 2), the fluorescence intensity produced by cleaved reporters shows a positive linear correlation with increasing reaction time (linear fit also shown, R^2 = 0.9828, n=2 independent reactions).

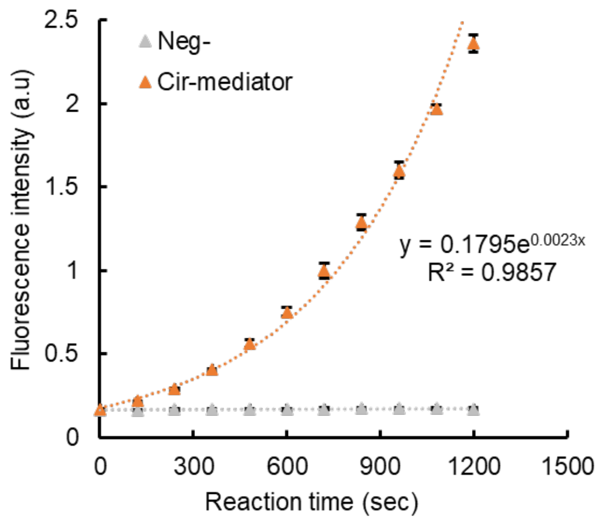

Supplementary Fig. 19 AutoCAR-1 *trans*-cleavage pattern. After the autocatalysis loop of AutoCAR-1 has been activated, the fluorescence signal intensity increased strongly with reaction time following a non-linear growth pattern, in response to addition of 1 pM ssDNA. "Neg-" represents an inactive AutoCAR-1 reaction mixture, without trigger ssDNA. (Method 7) Error bars represent mean ± SD, for n = 3 independent reactions.

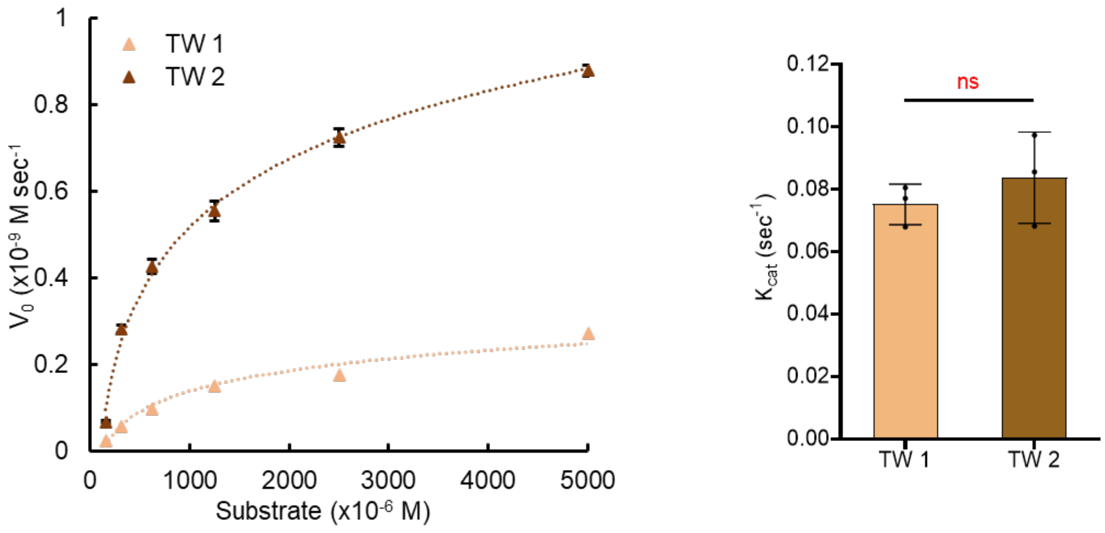

Supplementary Fig. 20 Michaelis-Menten analysis of the AutoCAR-1 *trans*-cleavage pattern in defined short reaction windows. The reaction rate is a nonlinear function of time, but in short time windows this function can be approximated as a linear time dependence. (A) Michaelis-Menten kinetics plot for the reporter cleavage rate in AutoCAR-1 with different concentrations of ssDNA reporters (substrates) and using an ssDNA trigger (n = 3 independent reactions). V_0 = the number of cleaved reporters per second, TW 1 = time window 1 (0-6 mins), TW 2 = time window 2 (20-30 mins). (B) Comparison of the estimated K_{cat} values of AutoCAR-1 in different reaction time windows. The data show no significant differences of the K_{cat} values which indicates that increased reporter cleavage rate in the AutoCAR-1 system is not attributable to changes induced in the Cas12a RNP *trans*-cleavage rate. (Methods 8, S7). Error bars represent mean ± SD, for n = 3 independent reactions.

(A)

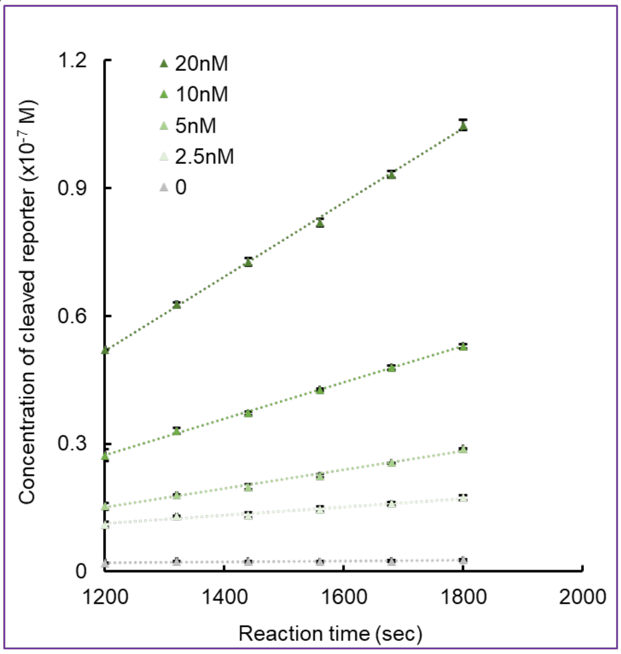

Slope of time window 2

(B)

20-30 mins	
Cir-mediator	Slope
20 nM	0.0009
10 nM	0.0004
5 nM	0.0002
2.5 nM	$1x10^{-4}$
0	$6x10^{-7}$

(C)

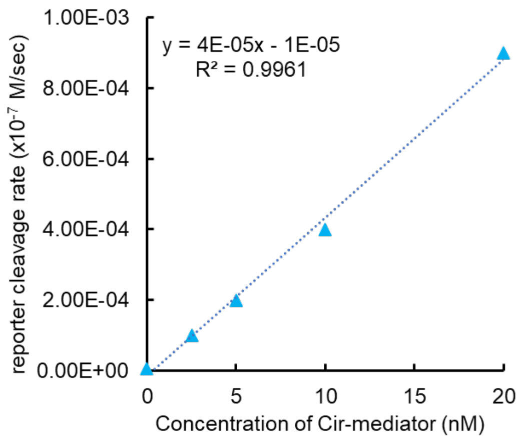

The reporter cleavage rate at time window 2

Supplementary Fig. 21 Reporter cleavage rate patterns of AutoCAR-1 in a specific short reaction time window. (A) We used the time window 2 in Fig. 4J, where a near linear signal trend with reaction time was found for different concentrations of Cir-mediators (n=3 independent reactions). (B) These reaction rates (slopes of linear fits in A) were used to estimate $R_{Cas12a/target}$ value of the AutoCAR-1 system. (C) Linear correlation between concentration of Cir-mediators and the reporter cleavage rate. Error bars represent mean ± SD, for n = 3 independent reactions. (Method 9).

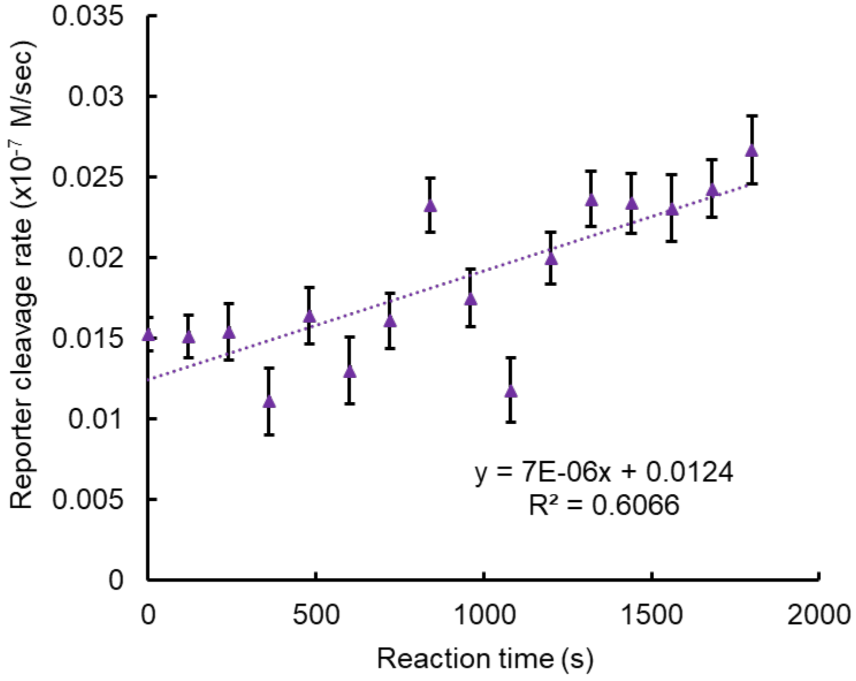

Supplementary Fig. 22 Reporter cleavage rate for standard CRISPR/Cas12a reaction without additional amplification in response to 1 pM trigger DNA. (Method 2) Error bars represent mean ± SD, for n = 3 independent reactions.

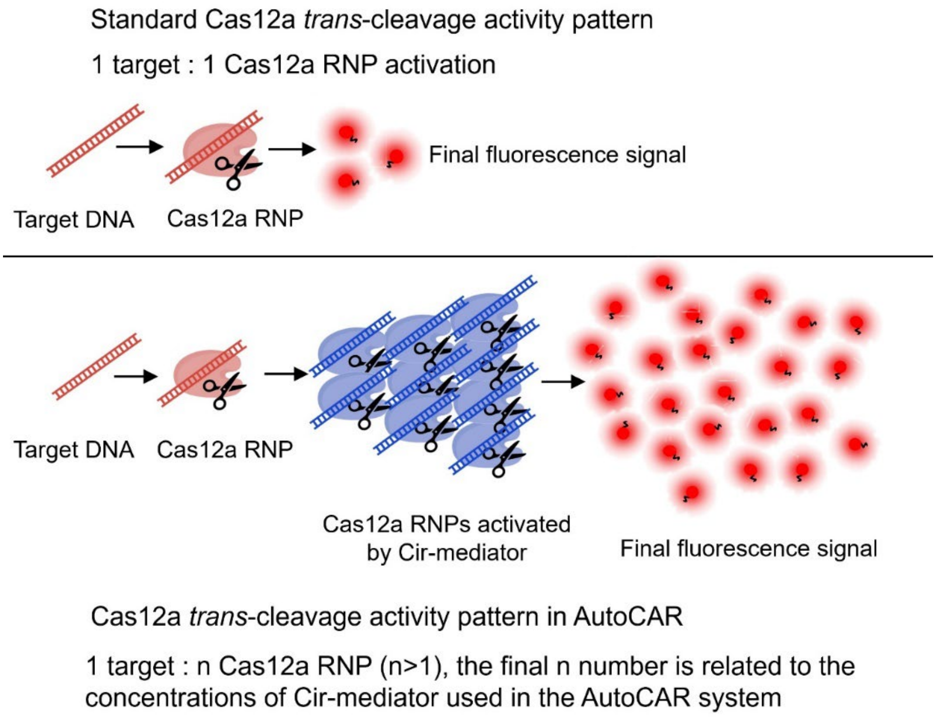

Supplementary Fig. 23 Schematics contrasting the reaction pattern for standard Cas12a RNP *trans*-cleavage (top) and the *trans*-cleavage pattern of the AutoCAR systems (bottom).

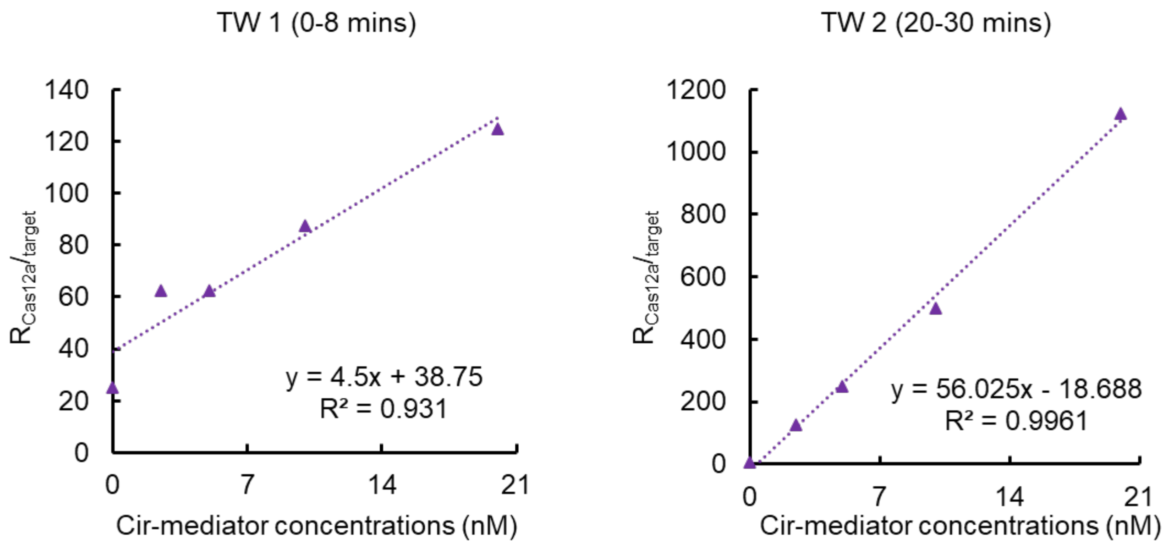

Supplementary Fig. 24 Correlation between the $R_{Cas12a/target}$ value (the number of overall activated Cas12a RNPs per a single trigger ssDNA) to the change of Cir-mediator concentrations in the AutoCAR-1 system. The results show that both for the reaction time window 1 (TW 1, 0-8 mins) and time window 2 (TW 2, 20-30 mins), there is a positive linear correlation between the $R_{Cas12a/target}$ value and the Cir-mediator concentrations in AutoCAR-1 system. This indicates that the concentration of activated Cas12a RNP is increased at higher concentration of Cir-mediator in the system, and its linear correlation confirms an increased level of Cas12a RNP activation in AutoCAR -1 system. (Method 9).

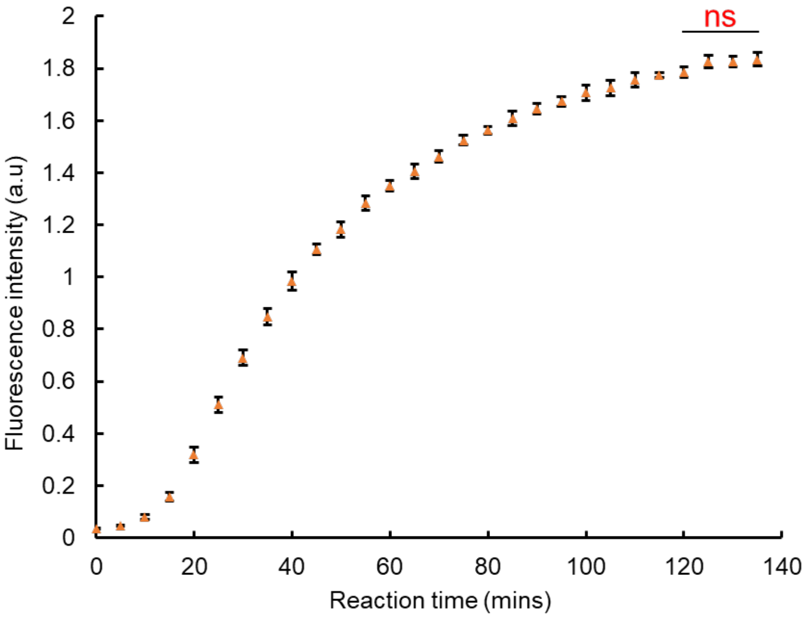

Supplementary Fig. 25 Depletion of reporters leads to saturation of the fluorescence signal. Illustration of signal trends in an AutoCAR reaction with a comparatively low concentration of ssDNA-linked fluorescence-quenched reporters. As Cas12a *trans*-cleavage continues over time, the fluorescence intensity level appears to saturate, with no significant changes after 120 mins (Method 7, with reporter concentration reduced to 39 nM). Error bars represent mean ± SD, for n = 3 independent reactions, ns=not significant.

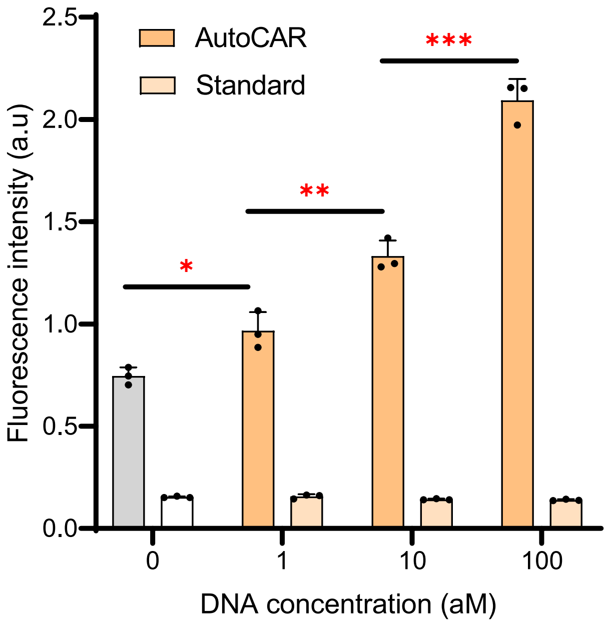

Supplementary Fig. 26 AutoCAR-1 system makes it possible for Cas12a RNP to reveal the presence of specific DNA sequence in the attomolar concentration range, down to 1 aM. While the standard CRISPR/Cas12a system without additional amplification strategy shows no detectable signal differences between the same target concentration ranges (Method S8). Brown bar represents AutoCAR reaction, light brown bar represents standard Cas12a reaction. Error bars represent mean ± SD, for n = 3 independent reactions, * $P<0.05$, ** $P<0.005$, *** $P<0.001$.

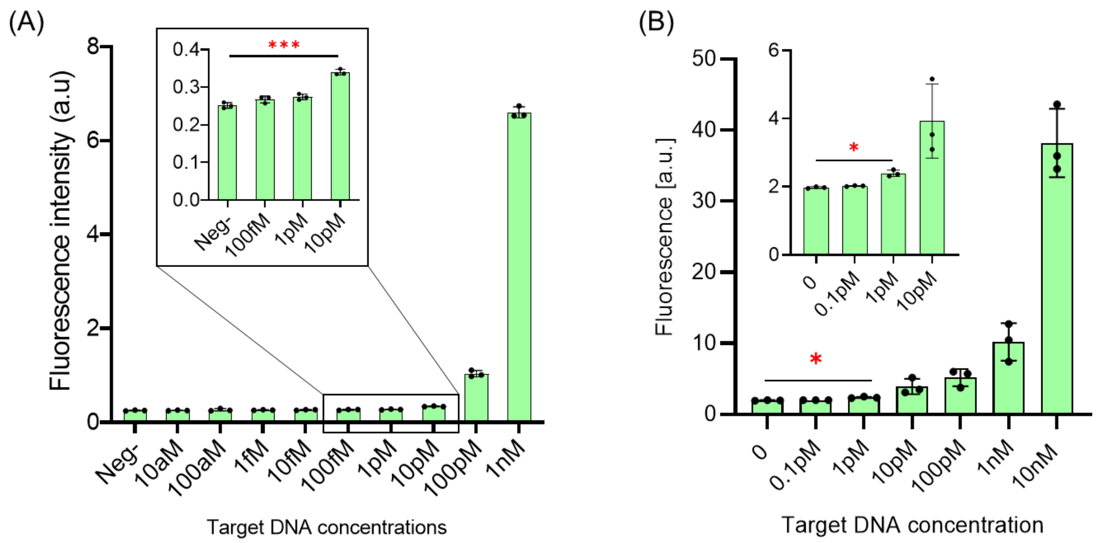

Supplementary Fig. 27 Reference data for standard CRISPR/Cas12a assays for direct DNA detection without the use of Cir-mediator-based autocatalysis. (A) The sensitivity is 10pM based on the statistical difference using two-tailed t-test (Method 2). (B) The sensitivity of a standard CRISPR/Cas12a biosensing system, which can reach 1pM (Method 14). Based on these tests, the detection limits for the standard CRISPR/Cas12a reaction are in the range of 1pM – 10pM. Error bars represent mean ± SD, for n = 3 independent reactions, * P<0.05, ** P<0.005, *** P<0.001.

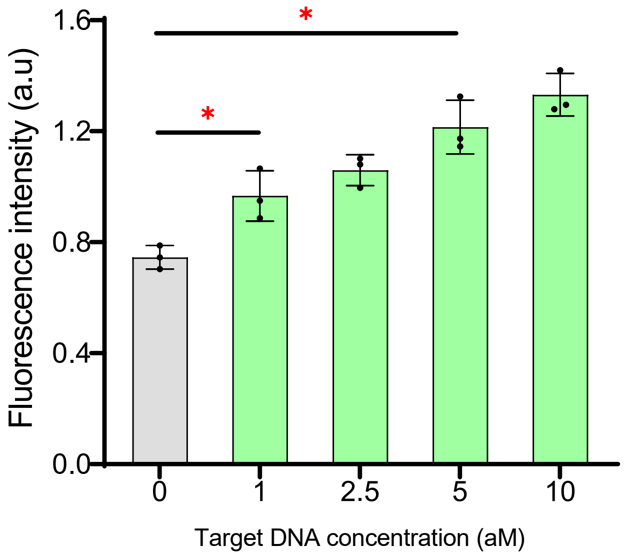

Supplementary Fig. 28 Performance of AutoCAR-1 for DNA detection at low concentration levels (Method S8). AutoCAR-1 system under these conditions is able to differentiate even small target concentration changes at low concentration level, here between 1 aM to 5 aM target DNA. Error bars represent mean ± SD, for n = 3 independent reactions, * P<0.05, ** P<0.005, *** P<0.001.

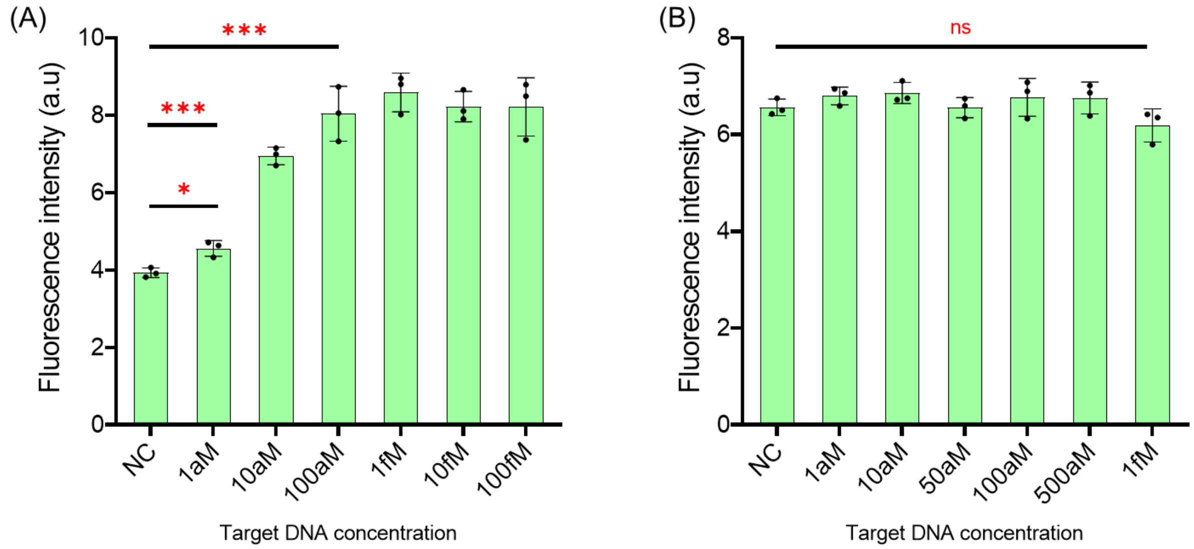

Supplementary Fig. 29 Performing AutoCAR-1 on a qPCR system in an ultra-sensitive DNA detection range (Method S9). (A) AutoCAR-1 system combined with detection of target DNA on a qPCR system. The results indicate that the AutoCAR-1 system can differentiate between a negative sample (NC) and samples with target DNA concentration between 1 aM to 100 aM. (B) Standard CRISPR/Cas12a reaction (without additional amplification strategy) to detect target DNA on a qPCR system; the results show no significant signal differences between a negative sample (NC) and any of the samples with target DNA between 1 aM to 1 fM on a qPCR system. Error bars represent mean ± SD, for n = 3 independent reactions, * $P<0.05$, ** $P<0.005$, *** $P<0.001$, ns=not significant.

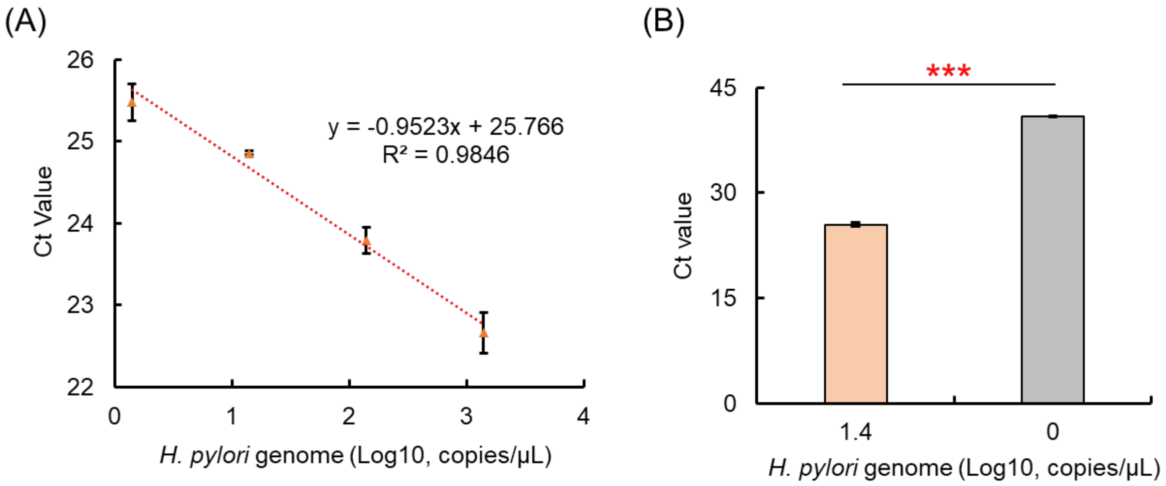

Supplementary Fig. 30 Quantitative PCR (qPCR) applied to *H. pylori glm* gene detection (Method S10). A negative linear correlation over 3 orders of magnitude has been identified in samples containing 1.4 to 1400 copies/µL *H. pylori* genome DNA, and qPCR is able to differentiate between negative sample (grey bar) and the presence of 1.4 copies/µL *H. pylori* genome DNA (brown bar). Error bars represent mean ± SD, for n = 3 independent reactions, * $P<0.05$, ** $P<0.005$, *** $P<0.001$.

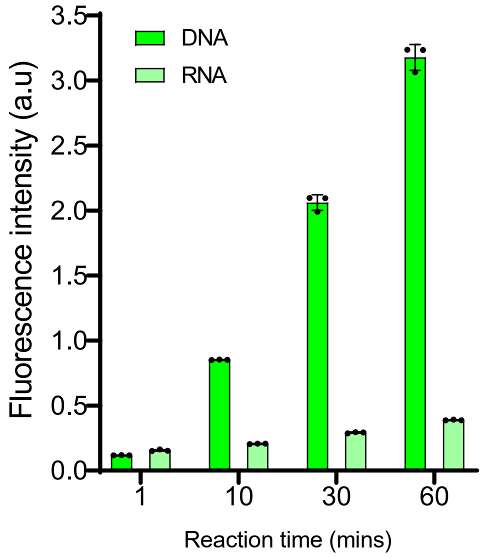

Supplementary Fig. 31 Difference of CRISPR/Cas12a *trans*-cleavage efficiency between DNA and RNA triggers detected by a standard CRISPR/Cas12a reaction without additional amplification strategy (Method 2). The results show that, comparing to DNA (ssDNA, green bar) whose fluorescence signal rapidly increases after Cas12a activation, RNA (ssRNA, light green bar) activates the Cas12a RNP at a significantly reduced level. Error bars represent mean ± SD, for n = 3 independent reactions.

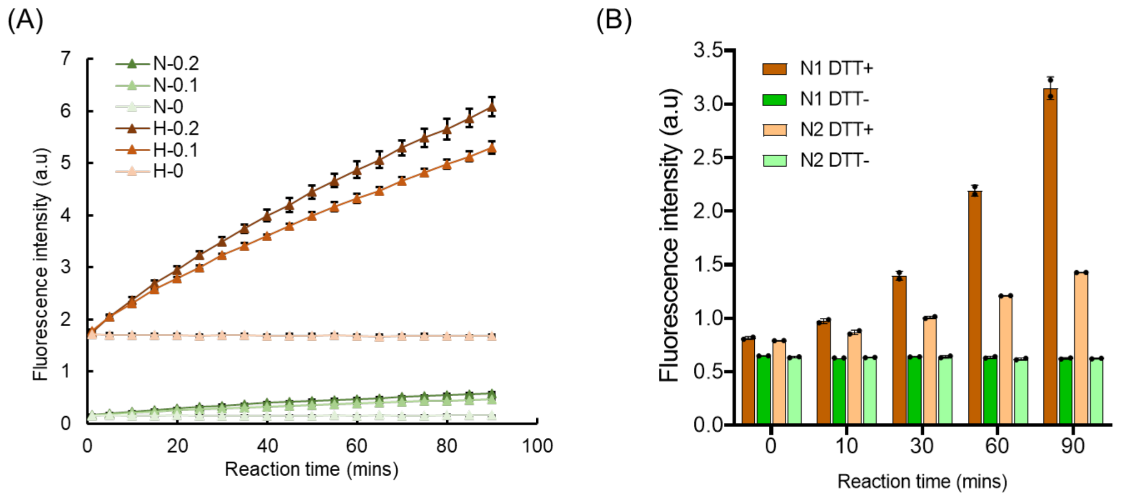

Supplementary Fig. 32 Optimization of the CRISPR/Cas12a *trans*-cleavage conditions for improved fluorescence signal generation in response to RNA trigger (Method S11). (A) CRISPR/Cas12a *trans*-cleavage pattern for RNA trigger (Supplementary Table 3, CoV-N-target. 0, 0.1, 0.2 μM) with different reporter concentrations (N = normal reporter concentration as in Method S11, H = 10 times higher reporter concentration). Error bars represent mean ± SD, for n = 3 independent reactions. (B) CRISPR/Cas12a *trans*-cleavage pattern for the RNA trigger with or without the use of DTT. N1 and N2 represent different Cas12a targeting sequences (Supplementary Table 3, Cas12a direct-N1 gRNA, Cas12a direct-N2 gRNA). The results indicate that the addition of 10mM DTT to a standard CRISPR/Cas12a reaction can significantly increase the signal output induced by the RNA target, and differences in targeted RNA sequences can also impact the output signal. Error bars represent mean ± SD, for n = 2 independent reactions.

(A)

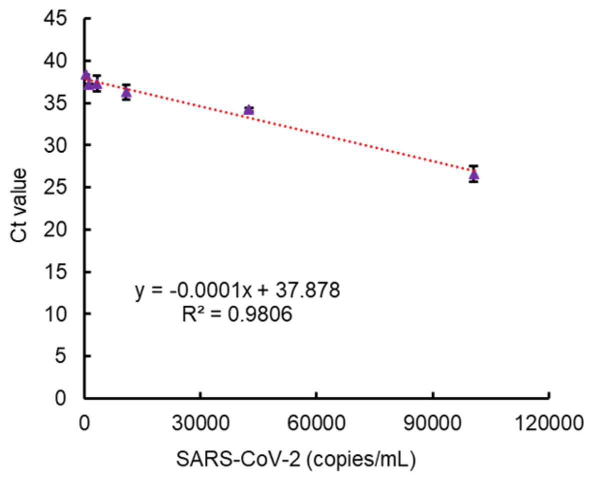

(B)

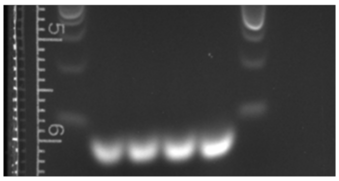

(C)

copies/mL	Ct Value	
	Sample 1	Sample 2
360	38.39	>40
1120	>40	37.21
3210	37.99	36.64
10750	35.70	36.93
42500	34.06	34.37
100610	27.23	25.97

Supplementary Fig. 33 Standard RT-qPCR method for SARS-CoV-2 genome RNA detection (Methods S12, S13). (A) The results show a typical RT-qPCR exhibits a linear correlation between the target RNA concentration and the Ct value (n=2 independent reactions). (B) Gel electrophoresis assay for RT-qPCR amplicon to verify the absence of non-specific amplification in the RT-PCR reaction system (Method 12). (C) The Ct value for different concentrations of SARS-CoV-2 genome RNA. In general, the cut-off Ct value representing a positive Covid-19 infection is between 35 to 40, and Ct value higher than 40 can be considered as a negative result. The results show the RT-qPCR performed here has the sensitivity between 360 and 3210 copies/mL, which corresponds to 0.36 ~ 3.21 copies/µL.

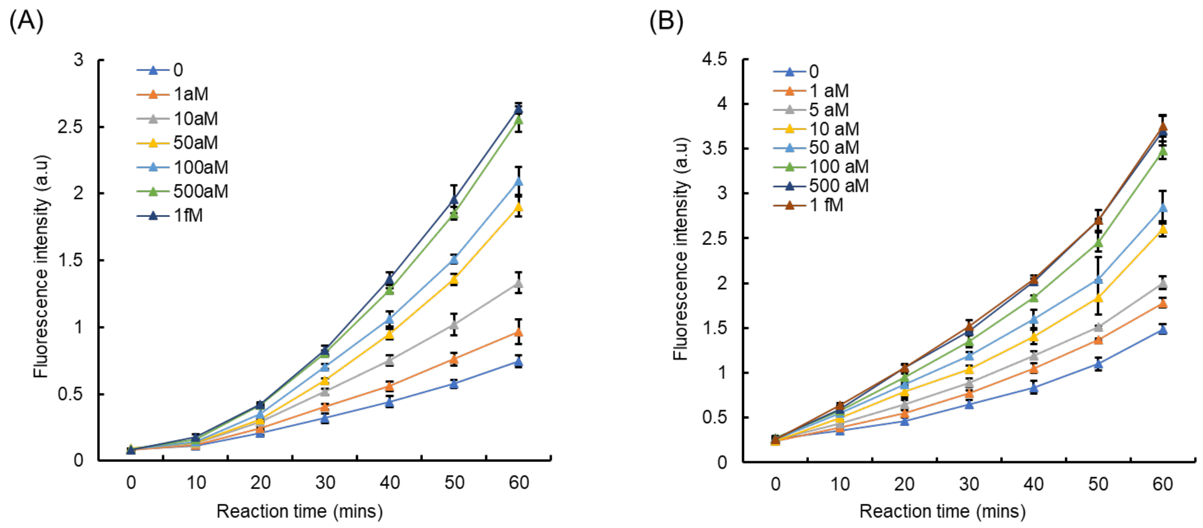

Supplementary Fig. 34 Kinetic fluorescence signal profiles for AutoCAR -1 detecting DNA and RNA. (A) AutoCAR-1 for DNA detection; (B) AutoCAR-1 for RNA detection (Method 7 & 11). Error bars represent mean ± SD, for n = 3 independent reactions.

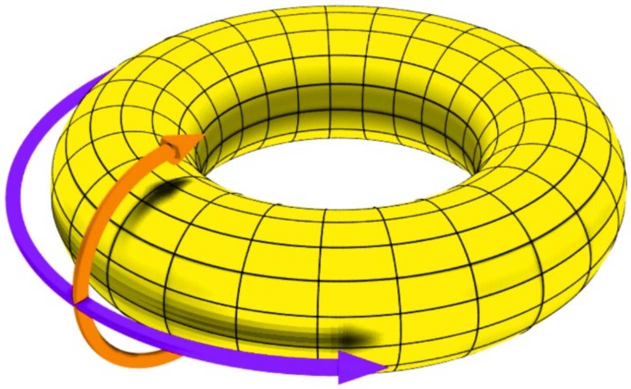

Supplementary Fig. 35 Idealised toroidal geometry of the Cir-mediator. Real Cir-mediator molecules are strictly speaking not toroidal, but closer to deformed toroids. Purple line indicates the direction along the length of the circle. The curvature radius of the purple line is small because the entire length of the Cir-mediator molecule along the purple line is ~ 7 nm. Orange line represents the “perpendicular” direction – this is the direction of rotation needed for releasing torsional stress during strand unwinding. This rotation is topologically constrained because the ends of the Cir-mediator molecule are joined together.

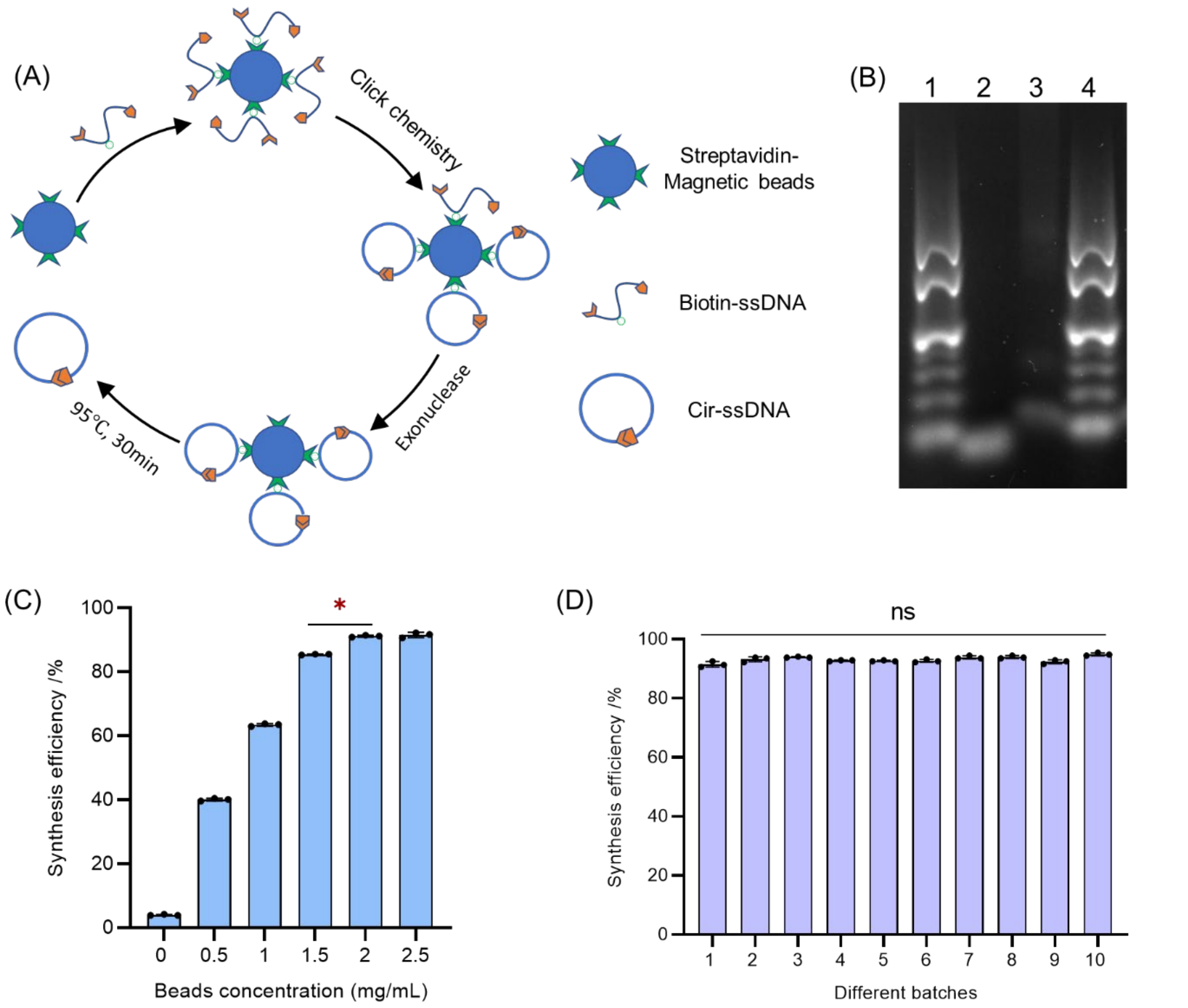

Supplementary Fig. 36 Synthesis and characterization of Cir-ssDNA (Method 13). (A) Schematic of the synthesis of Cir-ssDNA using magnetic bead-based click chemistry method. Biotin-ssDNA with specific modifications (5'-Azide (N3); 3'-CHCH; internal-Biotin), and the click chemistry approach was applied to form the Cir-ssDNA by bonding of azide and alkyne (CHCH) functional groups.[4, 5] The remaining linear ssDNA was degraded by exonuclease, and Cir-ssDNA was released from the streptavidin beads by heating to 95 °C.[6] (B) Demonstration of the formation of Cir-ssDNA using denaturing polyacrylamide gel (dPAGE) electrophoresis (from left to right :1. 10 bp ladder; 2. linear ssDNA; 3. Cir-ssDNA; 4. 10 bp ladder.), where the band of Cir-ssDNA (column 3) was found to move more slowly than the band of linear ssDNA (column 2), confirming the formation of Cir-ssDNA.[7] In addition, only a single band of Cir-ssDNA (column 3) was observed, consistent with the formation of single ring Cir-ssDNA. Thus, this magnetic bead-based click chemistry method improved the purity of single ring ssDNA synthesis. (C) Optimization of Cir-ssDNA synthesis efficiency using a bead-based click chemistry method, where synthesis efficiency of over 90% was achieved. (D) Reproducibility of Cir-ssDNA synthesis using a bead-based click chemistry method (coefficient of variation of 1.06%). Error bars represent mean ± SD, for n = 3 independent reactions, * P<0.05, ** P<0.005, *** P<0.001.

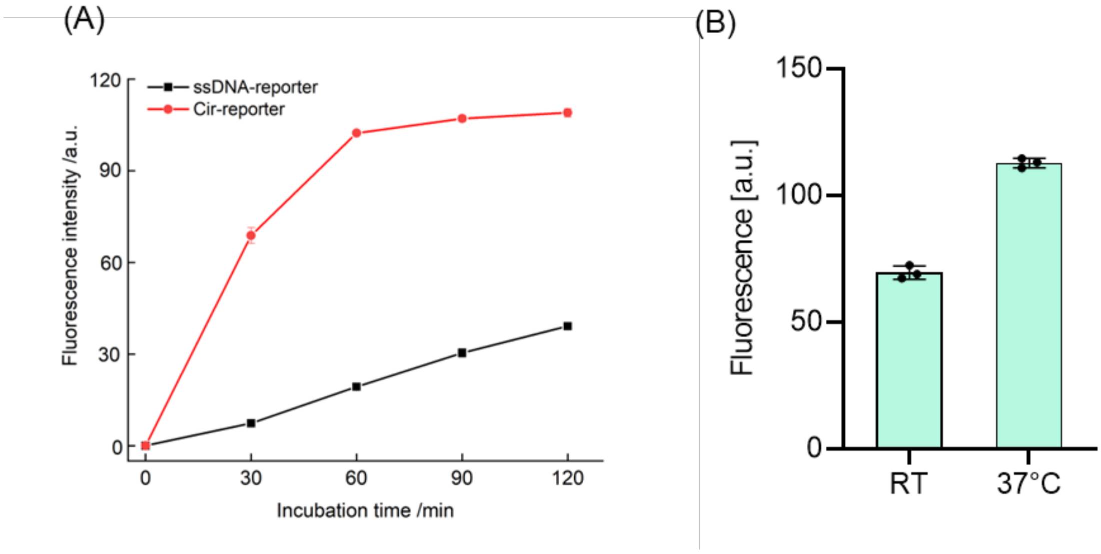

Supplementary Fig. 37 Evaluation of Cir-reporters as fluorescent reporters in a standard CRISPR/Cas12a biosensing system (Method 14). (A) Comparison of the biosensing performance of Cir-reporters and ssDNA reporter in a standard CRISPR/Cas12a biosensing system; (B) Comparison of the biosensing performance of Cir-reporter at RT and 37°C for 30 mins. Error bars represent mean ± SD, for n = 3 independent reactions.

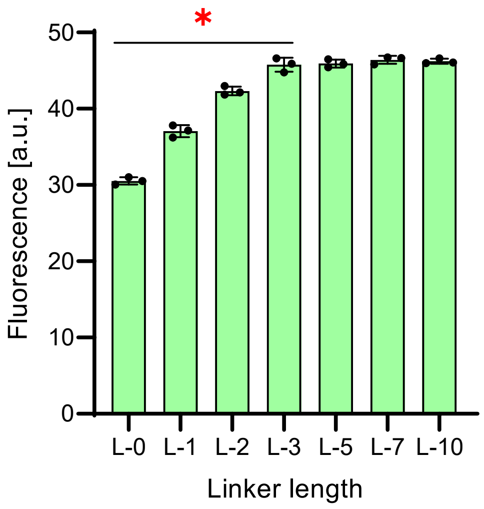

Supplementary Fig. 38 Investigation of the ssDNA linker length in the Cir-reporter (Method S14). The results show that a 3nt linker length is optimal. Error bars represent mean ± SD, for n = 3 independent reactions, * P<0.05, ** P<0.005, *** P<0.001.

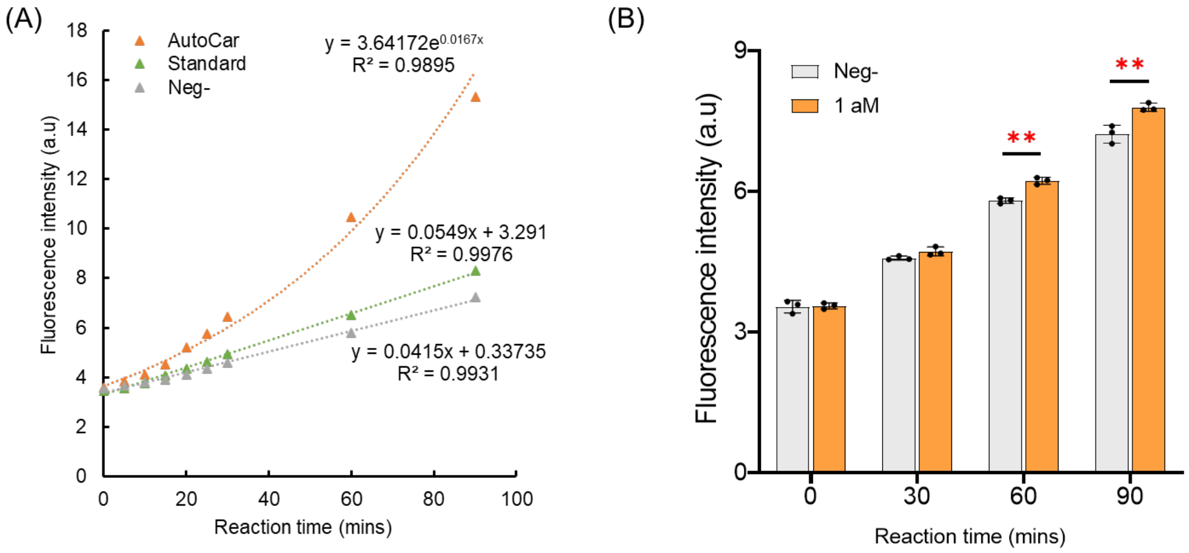

Supplementary Fig. 39 Establishment of AutoCAR-2 using AsCas12a protein (Method 17, which is the same method as used in Fig. 8). (A) Comparison of the biosensing performance of AsCas12a based AutoCAR-2 with a standard AsCas12a biosensing system, with trigger DNA concentration at 1 pM. (B) The limit of detection of AsCas12a based AutoCAR-2. Brown bar represents target concentration at 1 aM, grey bar represents the negative control. Error bars represent mean ± SD, for n = 3 independent reactions, * P<0.05, ** P<0.005, *** P<0.001.

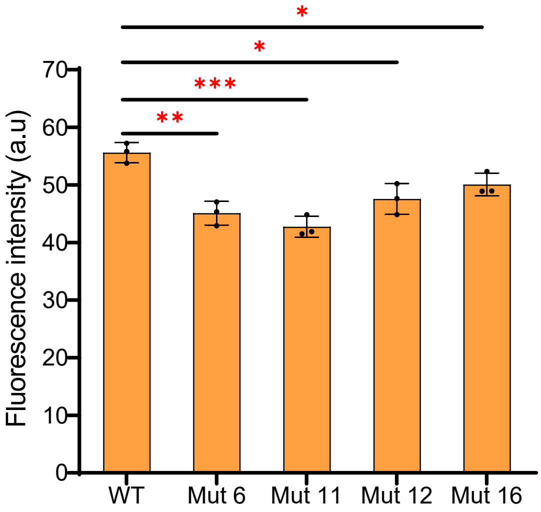

Supplementary Fig. 40 Differentiation of a single nucleotide mismatch in target dsDNA by AutoCAR-2. WT = dsDNA target fully matched to gRNA sequence of Cas12a RNP at 1 nM, Mut = single nucleotide mismatch located along the length of the target sequence from 5' to 3' at different loci of 6, 11, 12, 16, each at 1 nM. (n=3) (Method 17). Error bars represent mean ± SD, for n = 3 independent reactions, * $P<0.05$, ** $P<0.005$, *** $P<0.001$.

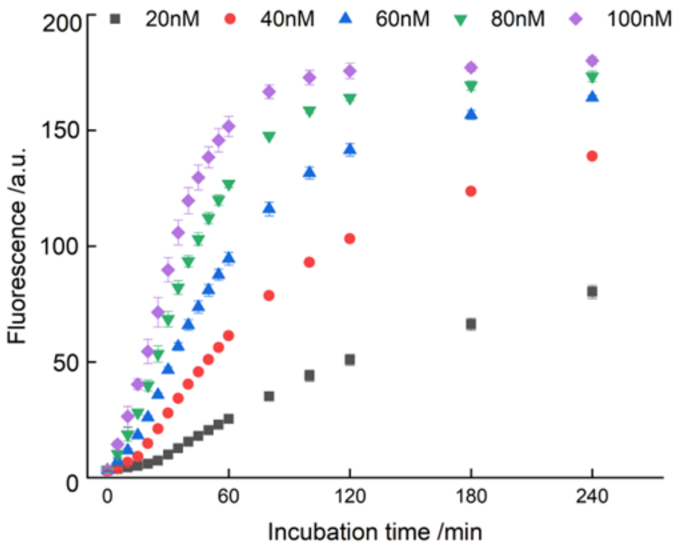

Supplementary Fig. 41 Illustration of signal time dependence in AutoCAR-2 in conditions where Cir-reporters are depleted in the course of reaction (Method S15). At long incubation times the fluorescence signal stabilizes reflecting the total number of cleaved reporters (and not targets). Error bars represent mean ± SD, for n = 3 independent reactions.

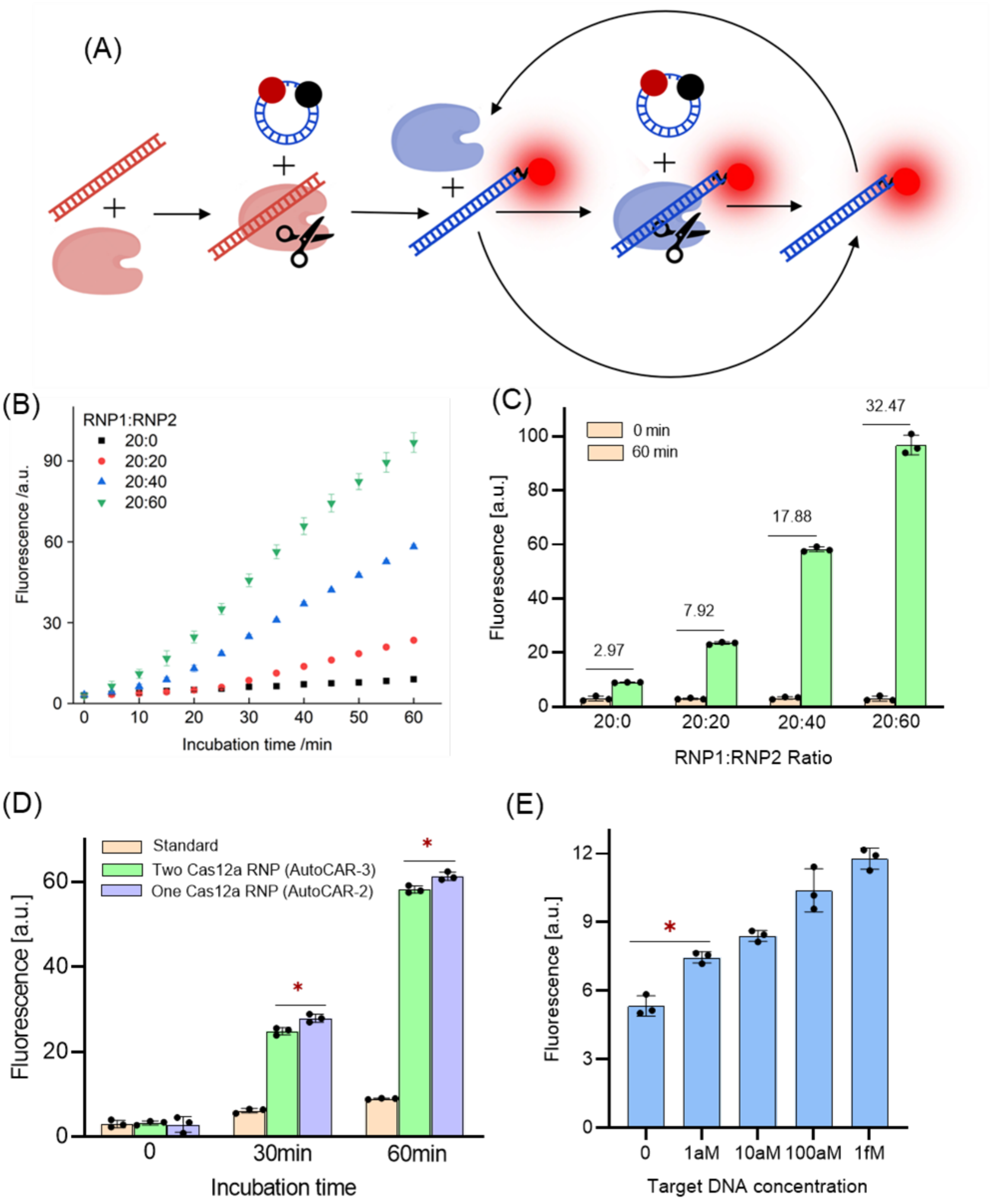

Supplementary Fig. 42 The development of Cir-reporter assisted autocatalysis biosensing system using two different types of Cas12a RNPs (AutoCAR-3) (Method S16). (A) Schematics ofAutoCAR-3; (B) The investigation of Cas12a RNP1 and RNP2 ratio (Cir-reporter 200 nM, and 1 pM target-C); (C) Amplified signals of Cas12a RNP1-RNP2 biosensing system (Cir-reporter 200 nM, and 1 pM target-C); (D) Comparison of AutoCAR-3 (two Cas12a RNPs, purple bar) and AutoCAR-2 (One Cas12a RNP, green bar), light brown bar represents a standard CRISPR/Cas12a reaction; (E) Limit of detection of AutoCAR-3. Error bars represent mean ± SD, for n = 3 independent reactions, * $P<0.05$, ** $P<0.005$, *** $P<0.001$.

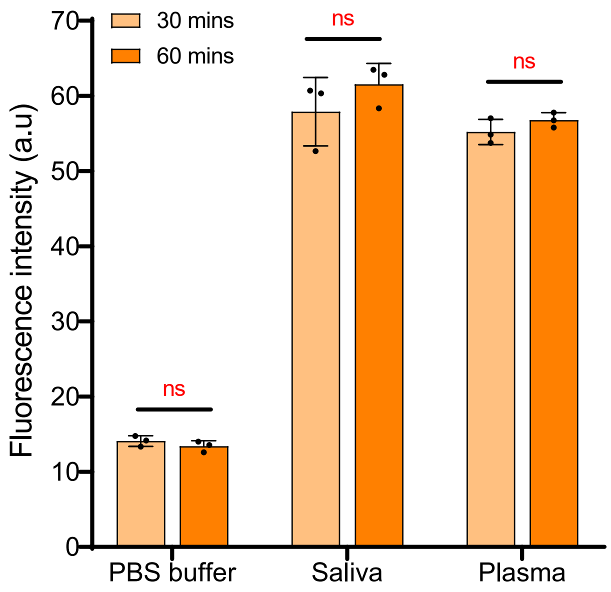

Supplementary Fig. 43 Stability test for circular DNA structures (Method S17, S18). The fluorescence signal generated from Cir-reporters remained statistically unchanged for 1 hour at room temperature, which indicates a stable structure of the circular DNA within thawed human saliva or plasma samples. Brown bar represents reaction results at 60 mins, light brown bar represents reaction results at 30 mins. Error bars represent mean ± SD, for n = 3 independent reactions, * $P<0.05$, ** $P<0.005$, *** $P<0.001$, ns=not significant.

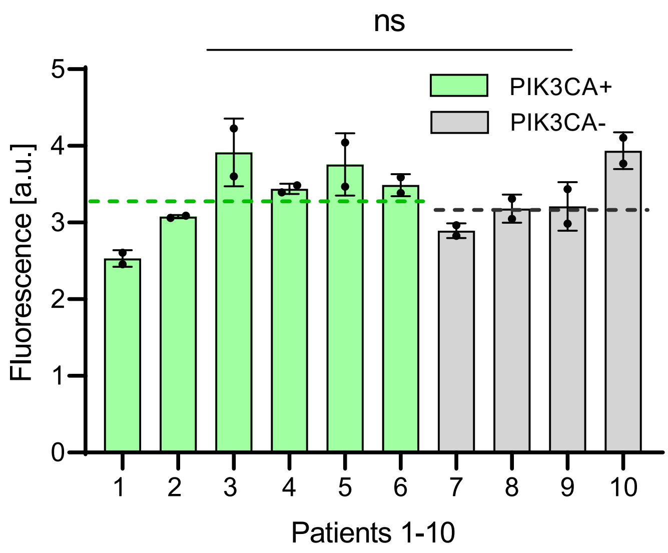

Supplementary Fig. 44 Background signal due to PIK3CA wild type gene fragments in patient plasma samples (Method 19). Here, the Cas12a RNPs are targeting the wild-type sequence of the PIK3CA gene in the region of the H1047R mutation. The wild-type sequence differs from the PIK3CA H1047R mutation sequence by a single nucleotide. The fluorescence signal for the PIK3CA H1047R + (green bar) and PIK3CA H1047R – (grey bar) patient groups shown here are indicative of the background signal level due to the wild type PIK3CA gene fragments in the tested plasma samples. The lack of significant difference between two patient groups confirms that our AutoCAR-3 system can specifically detect the PIK3CA H1047R mutation in ctDNA from the patient samples, as indicated in Fig. 9D. Error bars represent mean ± SD, for n = 2 independent reactions, * $P<0.05$, ** $P<0.005$, *** $P<0.001$, ns=not significant.

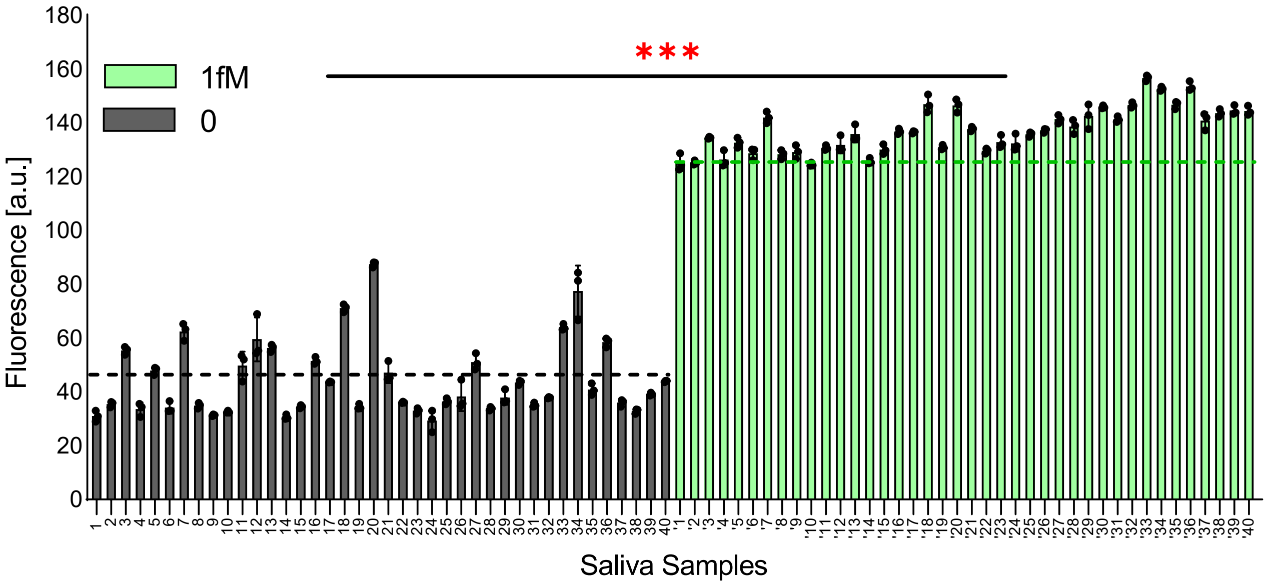

Supplementary Fig. 45 Feasibility of AutoCAR-3 detection in saliva (Method S17). N=40 saliva samples were tested as collected and after spiking with 1 fM of the PIK3CA H1047R mutation sequence. Saliva has undergone no preparation other than freezing at -20℃ degrees. Broken lines indicate averages of both groups (as collected and spiked). The data show statistical difference between these two groups (black group = without spiking, green group = spiked with 1fM of the PIK3CA H1047R mutation sequence). Error bars represent mean ± SD, for n = 3 independent reactions, *** P<0.001.

Supplementary Tables

Supplementary Table 1. Michaelis-Menten calculation for the *trans*-cleavage rates for AutoCAR-1 system at defined reaction time windows (Method S7).

Cleavage status	K_{cat} (sec^{-1})	K_m (M^{-1})	K_{cat}/K_m (sec^{-1} M^{-1})	V_{max}
Time window 1*	0.08 ± 0.01	2.05 ± 0.55 x 10^{-7}	3.67 x10^5	0.38 ± 0.04
Time window 2#	0.08 ± 0.02	1.87 ± 0.71 x 10^{-7}	4.40 x10^5	1.24 ± 0.24

* Time window 1 equals to 0-6 mins of the reaction time.
# Time window 2 equals to 20-30 mins of the reaction time.

Supplementary Table 2. The $R_{Cas12a/trigger}$ value (number of activated Cas12a RNP per target) of AutoCAR-1

Standard CRISPR/Cas12a without amplification (30 mins) *		Cir-mediator (nM)	AutoCAR at time windows (20-30 mins)	
Cleavage rate (x10^{-7}M/s)	$R_{Cas12a/trigger}$		Cleavage rate (x10^{-7}M/s)	$R_{Cas12a/trigger}$
7 x 10^{-6}	0.86	20	0.0009	1125
		10	0.0004	500
		5	0.0002	250
		2.5	0.0001	125

* As the standard CRISPR/Cas12a without amplification for 1 pM generated slightly increased fluorescence signal after 30 mins reaction, with shown a linear fit with R^2 = 0.61 only. Additional results (from Supplementary Fig. 18, 23) for standard CRISPR/Cas12a reactions without amplification have been utilised to verify the $R_{Cas12a/trigger}$ for standard CRISPR/Cas12a reaction, which has $R_{Cas12a/trigger}$ = 0.5.

Supplementary Table 3. DNA and RNA oligos used in this study (Fig. 1-5)

All the DNA and RNA oligonucleotides have been synthesized and modified by Sangon Ltd.

Name	Oligo type	Sequence 5'-3'	Modification
5'-P 15nt ssDNA	ssDNA	GTGCTATGTCTAAAA	5'- phosphorylation
5'-P 18nt ssDNA	ssDNA	T ATG TGC TAT GTC TAAA A	5'- phosphorylation
5'-P 19nt ssDNA	ssDNA	CT ATG TGC TAT GTC TAAA A	5'- phosphorylation
5'-P 20nt ssDNA	ssDNA	TCT ATG TGC TAT GTC TAAA A	5'- phosphorylation
5'-P 24nt ssDNA	ssDNA	TCAGTCTATGTGCTATGTCTAAAA	5'- phosphorylation
5'-P 27nt ssDNA	ssDNA	TTCTCAGTCTATGTGCTATGTCTAAAA	5'- phosphorylation
15nt cDNA	ssDNA	TTTAGACATAGCA	N/A
18nt cDNA	ssDNA	TTTAGACATAGCACAT	N/A
19nt cDNA	ssDNA	TTTAGACATAGCACATA	N/A
20nt cDNA	ssDNA	TTTAGACATAGCACATAG	N/A
24nt cDNA	ssDNA	TTTAGACATAGCACATAGACTG	N/A
27nt cDNA	ssDNA	TTTAGACATAGCACATAGACTGAGA	N/A
15nt linker ssDNA	ssDNA	ATAGCACTTTTA	N/A
18nt linker ssDNA	ssDNA	GCACATATTTTAG	N/A
19nt linker ssDNA	ssDNA	GCACATAGTTTTAG	N/A
20nt linker ssDNA	ssDNA	GCACATAGATTTTAG	N/A
24nt linker ssDNA	ssDNA	AGACTGATTTTAG	N/A
27nt linker ssDNA	ssDNA	CTGAGAATTTTAG	N/A
20nt ssDNA 3-Cy5	ssDNA	TCT ATG TGC TAT GTC **T**AAA A	5'- phosphorylation Int Cy5-dT
Trigger ssDNA	ssDNA	GAA GAC ACC CTA CCA ACC CCC CCC	N/A
ssDNA reporter	ssDNA	TTATT	5'-Texas Red; 3'-BHQ2
HP glm FW	ssDNA	TCC TTT TAG CGC TAC CGA CG	N/A
HP glm RV	ssDNA	CGA GCC ACA ACC CTT TTG AAG	N/A
CoV N FW	ssDNA	CAGAATGGAGAACGCAGTG	N/A
CoV N RV	ssDNA	GTGAACCAAGACGCAGTATT	N/A

Cir-T-strand gRNA	ssRNA	UAA UUU CUA CUA AGU GUA GAU GAC AUA GCA CAU AGA CUG AGA CUG	N/A
gRNA for FRET	ssRNA	UAA UUU CUA CUA AGU GUA GAU GAC AUA GCA CAU AGA CUG AGA CUG	5'- Cy3
Trigger ssDNA gRNA	ssRNA	UAA UUU CUA CUA AGU GUA GAU GGG GGG GGU UGG UAG GGU GUC	N/A
Cas12a direct-N1 gRNA	ssRNA	UAA UUU CUA CUA AGU GUA GAU ACG UUG UUU UGA UCG CGC CCC	N/A
Cas12a direct-N2 gRNA	ssRNA	UAA UUU CUA CUA AGU GUA GAU AUU GGG UAA ACC UUG GGG CCG A	N/A
CoV-N-target	ssRNA	CAGAA UGGAG AACGC AGUGG GGCGC GAUCA AAACA ACGUC GGCCC CAAGG UUUAC CCAAU AAUAC UGCGU CUUGG UUCAC	N/A

Supplementary Table 4. DNA and RNA oligos used in Fig. 6 and Supplementary Fig. 37. All the DNA and RNA oligonucleotides have been synthesized and modified by Sangon Ltd.

Name	Oligo type	Sequence 5’-3’	Modification
18nt cDNA	ssDNA	TTT AGA CAT AGC ACA TAG	5’-Texas Red; 3’-BHQ2
Cir-ssDNA-18nt (L-0)	ssDNA	CTA /iBio-dT/GT GCT ATG TCT AAA	5’-Azide (N3); 3’-CHCH
Cir-ssDNA-19nt (L-1)	ssDNA	T CTA /iBio-dT/GT GCT ATG TCT AAA	5’-Azide (N3); 3’-CHCH
Cir-ssDNA-21nt (L-3)	ssDNA	TTT CTA /iBio-dT/GT GCT ATG TCT AAA	5’-Azide (N3); 3’-CHCH
Cir-ssDNA-23nt (L-5)	ssDNA	TT TTT CTA /iBio-dT/GT GCT ATG TCT AAA	5’-Azide (N3); 3’-CHCH
Cir-ssDNA-25nt (L-7)	ssDNA	T TTT TTT CTA /iBio-dT/GT GCT ATG TCT AAA	5’-Azide (N3); 3’-CHCH
target-C	ssDNA	GAA GAC ACC CTA CCA ACC CCC CCC	N/A
gRNA-C	ssRNA	UAA UUU CUA CUA AGU GUA GAU GGG GGG GGU UGG UAG GGU GUC	N/A
ssDNA reporter	ssDNA	TTATT	5’-Texas Red; 3’-BHQ2

Supplementary Table 5. DNA and RNA oligos used in Fig. 7A

All the DNA and RNA oligonucleotides have been synthesized and modified by Sangon Ltd.

Name	Oligo type	Sequence 5'-3'	Modification
25nt cDNA	ssDNA	TTT AGA CAT AGC ACA TAG ACT GAG A	N/A
Cir-ssDNA-28nt	ssDNA	TTT TCT CAG TCT A/iBio-dT/G TGC TAT GTC TAA A	5'-Azide (N3); 3'-CHCH
21nt cDNA	ssDNA	TTT AGA CAT AGC ACA TAG ACT	N/A
Cir-ssDNA-24nt	ssDNA	TTT AGT CTA /iBio-dT/GT GCT ATG TCT AAA	5'-Azide (N3); 3'-CHCH
18nt cDNA	ssDNA	TTT AGA CAT AGC ACA TAG	N/A
Cir-ssDNA-21nt	ssDNA	TTT CTA /iBio-dT/GT GCT ATG TCT AAA	5'-Azide (N3); 3'-CHCH
15nt cDNA	ssDNA	TTT AGA CAT AGC ACA	N/A
Cir-ssDNA-18nt	ssDNA	TTT /iBio-dT/GT GCT ATG TCT AAA	5'-Azide (N3); 3'-CHCH
target-D	ssDNA	TCT CAG TCT ATG TGC TAT GTC	N/A
gRNA-D	ssRNA	UAA UUU CUA CUA AGU GUA GAU GAC AUA GCA CAU AGA CUG AGA	N/A
ssDNA reporter	ssDNA	TTATT	5'-Texas Red; 3'-BHQ2

Supplementary Table 6. DNA and RNA oligos used in Fig. 7B and Supplementary Fig. 38. All the DNA and RNA oligonucleotides have been synthesized and modified by Sangon Ltd.

Name	Oligo type	Sequence 5'-3'	Modification
18nt cDNA	ssDNA	TTT AGA CAT AGC ACA TAG	N/A
Cir-ssDNA-18nt (L-0)	ssDNA	CTA /iBio-dT/GT GCT ATG TCT AAA	5'-Azide (N3); 3'-CHCH
Cir-ssDNA-19nt (L-1)	ssDNA	T CTA /iBio-dT/GT GCT ATG TCT AAA	5'-Azide (N3); 3'-CHCH
Cir-ssDNA-20nt (L-2)	ssDNA	TT CTA /iBio-dT/GT GCT ATG TCT AAA	5'-Azide (N3); 3'-CHCH
Cir-ssDNA-21nt (L-3)	ssDNA	TTT CTA /iBio-dT/GT GCT ATG TCT AAA	5'-Azide (N3); 3'-CHCH
Cir-ssDNA-23nt (L-5)	ssDNA	TT TTT CTA /iBio-dT/GT GCT ATG TCT AAA	5'-Azide (N3); 3'-CHCH
Cir-ssDNA-25nt (L-7)	ssDNA	T TTT TTT CTA /iBio-dT/GT GCT ATG TCT AAA	5'-Azide (N3); 3'-CHCH
Cir-ssDNA-28nt (L-10)	ssDNA	T TTT TTT TTT CTA /iBio-dT/GT GCT ATG TCT AAA	5'-Azide (N3); 3'-CHCH

Supplementary Table 7. DNA and RNA oligos used in Fig. 8 and Supplementary Fig. 42

All the DNA and RNA oligonucleotides have been synthesized and modified by Sangon Ltd.

Name	Oligo type	Sequence 5’-3’	Modification
target-D	ssDNA	TCT CAG TCT ATG TGC TAT GTC	N/A
gRNA-D	ssRNA	UAA UUU CUA CUA AGU GUA GAU GAC AUA GCA CAU AGA CUG AGA	N/A
18nt cDNA	ssDNA	TTT AGA CAT AGC ACA TAG	5’-Texas Red; 3’-BHQ2
Cir-ssDNA-21nt	ssDNA	TTT CTA /iBio-dT/GT GCT ATG TCT AAA	5’-Azide (N3); 3’-CHCH
target-C	ssDNA	GAA GAC ACC CTA CCA ACC CCC CCC	N/A
gRNA-C	ssRNA	UAA UUU CUA CUA AGU GUA GAU GGG GGG GGU UGG UAG GGU GUC	N/A
ssDNA reporter	ssDNA	TTATT	5’-Texas Red; 3’-BHQ2

Supplementary Table 8. DNA and RNA oligos used in Fig. 9

All the DNA and RNA oligonucleotides have been synthesized and modified by Sangon Ltd.

Name	Oligo type	Sequence 5'-3'	Modification
H1047R WT Cis	ssDNA	GAA ACA AAT GAA TGA TGC ACA TCA TGG TGG CTG GAC AAC AAA AA	N/A
H1047R WT trans	ssDNA	TT TTT GTT GTC CAG CCA CCA TGA TGT GCA TCA TTC ATT TGT TTC	N/A
H1047R Mut Cis	ssDNA	GAA ACA AAT GAA TGA TGC ACG TCA TGG TGG CTG GAC AAC AAA AA	N/A
H1047R Mut trans	ssDNA	TT TTT GTT GTC CAG CCA CCA TGA CGT GCA TCA TTC ATT TGT TTC	N/A
H1047R Mut gRNA	ssRNA	UAA UUU CUA CUA AGU GUA GAU A UGA UGC ACG UCA UGG UGG	N/A
H1047R WT gRNA	ssRNA	UAA UUU CUA CUA AGU GUA GAU A UGA UGC ACA UCA UGG UGG	N/A
Cir-ssDNA	ssDNA	TTT A/iBio-dT/T TTA GAG AGA GGT AAA	5'-Azide (N3); 3'-CHCH
cDNA	ssDNA	TTT ACC TCT CTC TAA AAT	5'-Texas Red; 3'-BHQ2
cDNA-extend	ssDNA	TTT ACC TCT CTC TAA AAT CCC CC	5'-FAM; 3'-Biotin
ssDNA reporter	ssDNA	TTATT	5'-Texas Red; 3'-BHQ2

Supplementary Table 9. Summary and comparison of AutoCAR assay schemes introduced in this work.

**Assay feature**	**AutoCAR-1**	**AutoCAR-2**	**AutoCAR-3**	**AutoCAR-4**
Key purpose	Ultrasensitive nucleic acid assays with standard reporters	Ultrasensitive nucleic acid assays with standard reporters replaced by bespoke Cir-reporters	Amplification of conventional CRISPR assays	Ultrasensitive nucleic acid assays read by eye on a lateral flow test strip
name of DNA nanostructure	Cir-mediator	Cir-reporter	Cir-reporter	Cir-colour tag
Details of DNA nanostructure (all have PAM sequence)	dsDNA linked by a short ssDNA linker	dsDNA linked by a short ssDNA linker + fluorophore and quencher. Reporter DNA sequence is identical to target sequence	dsDNA linked by a short ssDNA linker + fluorophore and quencher	dsDNA linked by a short ssDNA linker + FAM +biotin on an ssDNA tail
Matching of dsDNA to target sequence	Assay will work with and without matching (only the case of no matching is presented here)	Matching	No matching	Assay will work with and without matching (only the case of no matching is presented here)
DNA nanostructure schematics	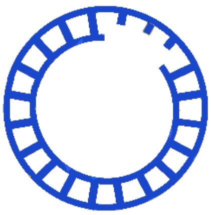	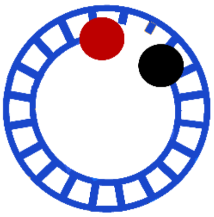	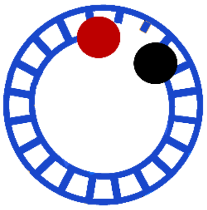	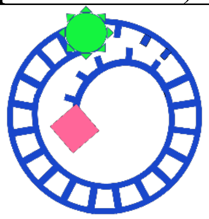
Details of the assay scheme	Fig. 1 & 4	Fig. 6	Supplementary Fig. 42	Fig. 9
Single pot reaction	Yes	Yes	Yes	Yes, but followed by LFA
Key advantage (beyond setting up autocatalysis)	Suitable as a standalone assay.<br><br>Can amplify third party CRISPR sensors.	No competition between Cir-mediators powering autocatalysis and standard reporters for the available *trans-*	No competition between Cir-mediators powering autocatalysis and standard reporters for the available *trans-*	Enables colorimetric readout, but in itself is not a colorimetric reporter.

		cleavage, hence assays can be faster.	cleavage<br><br>Suitable as a standalone assay.<br><br>Suitable to amplify third party CRISPR sensors	

Supplementary Table 10. List of abbreviations

AutoCAR	Autocatalytic CRISPR/Cas12a Amplification Reaction
c-DNA	Complementary DNA
Cir-ssDNA	circular single strand DNA
ss-DNA	single strand DNA
dsDNA	double strand DNA
FRET	fluorescence resonance energy transfer assay
gRNA	guiding RNA or presenting the crRNA (CRISPR RNA), sgRNA (single guiding RNA)
L-dsDNA	linear double strand DNA
L-ssDNA	linear single strand DNA
PAM	protospacer adjacent motif
RNP	ribonucleoprotein
ssDNA	single strand DNA
target-C	target DNA for classic CRISPR/Cas12a sensor
gRNA-C	gRNA for classic CRISPR/Cas12a sensor
target-D	target DNA for AutoCAR-2
gRNA-D	gRNA for AutoCAR-2
ctDNA	circulating tumor DNA
gRNA-ct	gRNA used for ctDNA detection
CRC-mouse	mouse bearing human colorectal cancer
LFA	Lateral flow assay
PCR	Polymerase Chain Reaction
RPA	Recombinase polymerase amplification
LAMP	Loop-mediated isothermal amplification
SERS	Surface-enhanced Raman spectroscopy
LOD	Limit of detection

Supplementary Methods

Materials

T4 ligase (NEB), T4 ligase buffer (NEB), exonuclease III (NEB), LbCas12a (NEB), NEB2.1 buffer (NEB), agarose (ThermoFisher), TBE buffer, Urea-PAGE (Bio-rad), SYBR Gold DNA dye (ThermalFisher), 100 bp DNA ladder (ThermoFisher), 10 bp DNA ladder (ThermoFisher), 6X DNA loading dye (ThermoFisher), DTT (ThermoFisher), QuantiNova SYBR Green PCR kit (QIAGEN, 208052), QuantiNova SYBR Green RT-PCR kit (QIAGEN, 208152), AMPLIRUN *Helicobacter Pylori* DNA control (Vircell, MBC049-R, NCBI No. NC_000915.1), deactivated SARS-CoV-2 viral particles (Certified Reference Materials, National Measurement Institute, Australia).

Chemicals and biological reagents: EnGen® Lba Cas12a (Cpf1) protein (New England Biolab), 10X NEB 2.1 buffer (New England Biolab), exonuclease VII (New England Biolab), SYBR Gold DNA dye (ThermoFisher), 10 bp DNA ladder (ThermoFisher), 6X DNA loading dye (ThermoFisher), copper sulfate ($CuSO_4$) (Sigma, 209198), Tris(2-carboxyethyl) phosphine (TCEP) (Sigma, C4706), tris(benzyltriazolylmethyl) amine (TBTA) (ChemSupply, T2993), streptavidin coated magnetic particles (Spherotech, SVM-08-10), DNase/RNase free water (ThermoFisher), phosphate buffered saline (PBS) (Sigma, 10 mM, pH=7.4), and HybriDetect – Universal Lateral Flow Assay Kit (Millenia Biotec, all antibodies pre-implemented on the commercialized lateral flow strip).

All DNA and RNA oligos are synthesized and modified by Sangon Bio-Tech Ltd.

Method S1. Synthesis of Cir-ssDNA with diluted ligase buffer at different rates

Firstly, 43 µL of DNase/RNase free water was mixed with different volumes (5, 1, 0.5, 0.25, 0.1 µL) of 10X T4 ligase buffer to form the final dilution times (including 1X, 0.2X, 0.1X, 0.05X and 0.02X), and followed with the adding of 2 µL of the linear ssDNA oligo (100 µM), 4 µL of linker ssDNA oligo (100 µM) and 2.5 µL of T4 ligase (NEB) to form a final 50 µL reaction mixture. The cyclization reaction was then allowed to proceed at 16°C for 12 hours and then deactivated at 65°C for 10min. The products were stored at 4°C before downstream applications.

Method S2. Removing residual linear ssDNA from Cir-ssDNA synthesis product

The Cir-ssDNA was synthesized by mixing 2 µL of 20 nt linear ssDNA (100 µM), 4 µL of linker ssDNA oligo (100 µM), 2.5 µL of T4 ligase (NEBuffer), 1 µL of T4 ligase buffer, and 43 µL of DI water, and incubated at 16℃ for 12 hours and then 65℃ for 10min. Then, the un-ligated linear ssDNA and the linker ssDNA was depredated with a mixture containing 1.5 µL of exonuclease III, 10 µL of Cir-ssDNA product in 40 µL of 1×NEBuffer 2.1 buffer, and incubation at 37°C for 100 mins followed with deactivation of exonuclease III at 75°C for 30 mins.

Method S3. **Validation of Cas12a *trans*-cleavage for ssDNA and dsDNA degradation following activation of Cas12a RNP2**

1 μL of 100 μM LbaCas12a endonuclease (NEB, M0653T) and 5 μL of 20 μM gRNA was mixed at 3.6 mL 1× NEBuffer 2.1, and followed with the adding of 1 μM L-ssDNA or L-dsDNA. Then, 10 μL of 1 μM trigger ssDNA was mixed with 90 μL of the prepared standard CRISPR/Cas12a reaction mixture. The reaction was set at room temperature, and each of 10 μL cleavage product from 0, 10, 20, 30, 40, 50, 60 mins was mixed with 2 μL 6X DNA gel loading dye, and then loaded onto 4% agarose gel for electrophoresis at a constant voltage of 80V for 60 min. Gel images were visualized by using Gel Doc + XR image system (Bio-Rad Laboratories Inc., USA).

Method S4. Using different concentrations of *trans*-cleaved Cir-mediator for Cas12a RNP2 detection

1 μL of 100 μM LbaCas12a endonuclease (NEB, M0653T) and 5 μL of 20 μM gRNA was mixed at 3.6 mL 1× NEBuffer 2.1, and followed with the adding of 100 nM of prepared DNA oligo (linear dsDNA, linear ssDNA, Cir-ssDNA or Cir-mediator). Then, 1 μM of trigger ssDNA oligo was added to activate Cas12a *trans*-cleavage for 60 mins at room temperature. Subsequently, heat all the reaction solutions at 65 °C for 10min to deactivated Cas12a RNP1. Afterwards, 10 μL of the *trans*-cleavage product was transferred into 90 μL of the standard CRISPR/Cas12a reaction mixture with gRNA for Cir-mediator, and set at room temperature for 60 mins. The fluorescence intensity at *E*x/*E*m of 570/615 nm was determined by using a plate reader (iD5 Spectramax, Molecular Devices, USA).

Method S5. FRET efficiency test for the Cy3-Cy5 pair used in this study

The Cy5 labelled ssDNA oligo was firstly mixed with its cDNA to form dsDNA, then 5 μL 1 μM of the prepared DNA oligo was mixed with 95 μL of 1×NEBuffer 2.1 containing Cy3 labelled gRNA loaded Cas12a RNP to a final concentration of 50 nM for both the DNA oligo and Cas12a RNP. The fluorescence signals were then collected by using a plate reader (iD5 Spectramax, Molecular Devices, USA) as *E*x= 520 nm and *E*m= 560 nm for acquiring *F*d, and *E*x= 520 nm and *E*m= 666 nm for acquiring *F*a.

Method S6. **Exploring the AutoCAR-1 reaction signal intensity with Cir-mediator concentration changes**

3 μL of 100 μM LbaCas12a endonuclease (NEB, M0653T), 5 μL of 20 μM gRNA1 (for trigger DNA/RNA) and 10 μL of 20 μM gRNA2 (for Cir-mediator) was mixed at 3.6 mL 1× NEBuffer 2.1 and followed with the adding of 12 μL of 100 μM Texas Red quenched reporter. Afterwards, the Cir-mediator solution was mixed to a final concentration of 0, 5, 12.5, 25 and 50 nM. Then, 10 μL 10 pM trigger ssDNA was mixed with 90 μL of the prepared final AutoCAR reaction mixture to initiate the reaction. The reaction was set at room temperature and the fluorescence intensity at *E*x/*E*m of 570/615 nm was determined by using a plate reader (iD5 Spectramax, Molecular Devices, USA).

Method S7. Calculation of *trans*-cleavage rates for AutoCAR-1 system at defined reaction time windows

Based on the results from Fig. 4E, the reaction of AutoCAR with difference concentration of reporters (156 nM to 5 uM) can be treat as two reaction time windows, the first window (0-6 mins) where the reaction begin with limited Cir-mediator has been prelinearized, and the second time window where majority of Cir-mediator has been depleted. For these two time windows, an approximately linear correlation can be acquired (Fig. 4E), therefore, this supports the application of basic Michaelis-Menten theory in these defined two time windows to estimate the observed *trans*-cleavage rates using the previously published method [8]. Briefly, the data of two time windows (TW1, TW2) at each reporter concentrations was fitted using linear regression to obtain the slope as initial reaction velocities, and plotting it against the substrate (reporter) concentration to fit to the Michaelis-Menten equation using Origin software (Origin, MA, US) to obtain V_{max} and K_m.

Method S8. Investigations of AutoCAR-1 sensitivity

3 µL of 100 µM LbaCas12a endonuclease (NEB, M0653T), 5 µL of 20 µM gRNA1 (for trigger DNA/RNA) and 10 µL of 20 µM gRNA2 (for Cir-mediator) was mixed at 3.6 mL 1× NEBuffer 2.1 and followed with the adding of 12 µL of 100 µM Texas Red quenched reporter. Afterwards, the prepared Cir-mediator solution was mixed to a final concentration of 50 nM before use to form the final AUTOCAR reaction mixture. Then, 10 µL different concentrations of trigger DNA with a complementary sequence to gRNA was mixed with 90 µL of the prepared final AUTOCAR reaction mixture to initiate the reaction. The reaction was set at room temperature and the fluorescence intensity at *E*x/*E*m of 570/615 nm was determined by using a plate reader (iD5 Spectramax, Molecular Devices, USA).

Method S9. Using AutoCAR-1 for DNA diagnostics on a qPCR thermocycler

6 µL 100 µM of LbaCas12a endonuclease (NEB, M0653T) was mixed with 30 µL 20 µM of gRNA1 (for target DNA) and 30 µL 20 µM of gRNA2 (for Cir-mediator) within 1.2 mL of 1×NEB2.1 buffer. Then, 2.4 µL 100 µM of Texas Red reporters was added. Before the DNA detection test, 5 µL of 500nM Cir-mediator solution were firstly mixed with 40 µL of the prepared reaction mixture, and 5 µL samples were added containing different concentrations of the target DNA sequence. The reaction was then set at 37°C on a CFX96 Touch Real-Time PCR Detection System (Bio-Rad Laboratories Inc., USA). Then fluorescence intensity was determined by the selection of the Texas Red channel of the thermocycler in 30 sec intervals.

Method S10. The qPCR reaction for *H.pylori glm* gene detection

The manufacturer's instruction of QuantiNova SYBR Green PCR kit (QIAGEN, 208052) was used for the qPCR reactions. Briefly, for each 20 µL reaction mixture, 10 µL 2X SYBR master mixture, 2 µL 10 µM Forward-primer and 2 µL 10 µM Reverse-primer were mixed with 4 µL of DNase/RNase free water. Then, 2 µL of sample with difference concentrations of *H.pylori* genome DNA (0, 1.4, 14, 140, 1400, 14000 copies/µL) was added to form the final 20 µL reaction mixture. Then qPCR reaction was set on a CFX96 Touch Real-Time PCR Detection System (Bio-Rad Laboratories Inc., USA) at the conditions of 95°C 2 mins, and followed with 35 cycles of amplification at 95°C 10 sec and 60°C 20 sec. The default melting curve analysis was also added for validation of the RT-qPCR.

Method S11. **Optimization of CRISPR/Cas12a RNP response to RNA trigger for higher signal generation**

1 μL of 100 μM LbaCas12a endonuclease (NEB, M0653T) and 5 μL of 20 μM gRNA was mixed at 3.6 mL 1× NEBuffer 2.1, and followed by adding of 6 μL or 60 μL of 100 μM Texas Red quenched reporter. In addition, the reaction mixture can be prepared with or without adding of DTT to a final concentration of 10 mM. For each CRISPR/Cas12a *trans*-cleavage activation reaction, 10 μL different concentrations of RNA with complementary sequence of gRNA was added into 90 μL prepared reaction buffer. The reaction was set at room temperature, and the fluorescence intensity at *E*x/*E*m of 570/615 nm was determined by using a plate reader (iD5 Spectramax, Molecular Devices, USA).

Method S12. **RNA extraction from SARS-CoV-2 viral particles**

The PureLink Viral RNA/RNA Mini Kit was used to extract the genome RNA of SARS-CoV-2 viral particles (Certified Reference Materials from National Measurement Institute) following the manufacturer's instruction. Briefly, 200 μL of viral sample was mixed with 25 μL Proteinase K (included with the kit), and followed with the adding of 200 μL Lysis Buffer containing 5.6 μg Carrier RNA (included with the kit). The mixture was then incubated at 56°C for 15 mins. After a briefly centrifuge, 250 μL 100% ethanol was added to the mixture and vortexing for 15 sec. The mixture was incubated at room temperature for 5 mins and then transferred onto the Viral Spin Column for centrifugation at 6800 x g for 1 min. The flow-through was discard, and followed with 2 times of washing with 500 μL prepared Wash Buffer and centrifugation at 6800 x g for 1 min. Afterwards, Centrifuge the column at maximum speed for 1 mins to dry the membrane completely. Then, 25 μL of Sterile, RNase free water was loaded onto the centre of the membrane and incubate at room temperature for 1 min. After centrifugation again at maximum speed again for 1 min, the eluted purified RNA was stored at -20°C immediately until the downstream application.

Method S13. **RT-qPCR reaction for SARS-CoV-2 RNA N-gene detection**

The manufacturer's instruction of QuantiNova SYBR Green RT-PCR kit (QIAGEN, 208152) was used for the RT-qPCR reactions. Briefly, for each 20 μL reaction mixture, 10 μL 2X SYBR mixture, 0.2 μL reverse transcription mixture, 1 μL 10 μM Forward-primer and 1 μL 10 μM Reverse-primer were mixed with 5.8 μL of DNase/RNase free water. Then, 2 μL of sample with different concentrations of extracted SARS-CoV-2 genome RNA (0, 360, 1120, 3210, 10750, 42500, 100610 copies/mL, Method S11) was added to form the final 20 μL reaction mixture. The RT-qPCR reaction was set on a CFX96 Touch Real-Time PCR Detection System (Bio-Rad Laboratories Inc., USA) at the conditions of 50°C 10mins and 95°C 3 mins, and followed by 40 cycles of amplification at 95°C 10 sec and 60°C 20 sec. The default melting curve analysis was also added for validation of the RT-qPCR.

Method S14. **Investigation of the ssDNA linker length in Cir-reporter**

To investigate the ssDNA linker length in Cir-reporter, we conducted the following two-step experiments. In the first step, the reaction mixture was prepared as follows: 1 μL 100 μM (100 pmol) of Cas12a protein was gently mixed with 5 μL 20 μM (100 pmol) of gRNA-C (Supplementary Table 7) in 3.6 mL 1X NEB 2.1 buffer. Then, 180 μL of 5 μM (0.9 nmol) of Cir-reporter with different linker length were added and well mixed to form the reaction mixture. Afterwards, 10 μL 0.25 μM of the target-C ssDNA was added into 90 μL of the prepared CRISPR/Cas12a reaction mixture for activating *trans*-cleavage of Cas12a and

cleaving Cir-reporters to linearized Cir-reporters. After one hour incubation at room temperature, the reaction mixture was collected for further use. To eliminate the influence of activated Cas12a RNPs from step 1, the reaction mixture was heated to 65 °C for 10 min to deactivate all the Cas12a RNPs from step 1. This ensures that only linearized Cir-reporter will be the active trigger for downstream step 2 biosensing system.
In the second step, the reaction mixture was prepared as follows: 1 μL 100 μM (100 pmol) of Cas12a protein was gently mixed with 5 μL 20 μM (100 pmol) of reporter-gRNA in 3.6 mL 1X NEB 2.1 buffer. Then, 6 μL of 100 μM (0.6 nmol) of pre-synthesized fluorescent quenched ssDNA reporters (Texas red-TTATT-BHQ2) were added and well mixed to form the reaction mixture. Subsequently, 10 μL of prepared reaction mixture from step 1 (25 nM) was added to 90 μL CRISPR/Cas12a reaction mixture prepared in step 2 and incubated for 120min. A SpectraMax iD5 multi-Mode Microplate Reader (Molecular Devices) was applied for the detection of fluorescence readout. The *E*x/*E*m of Texas red-TTATT-BHQ2 reporter was 570/615 nm.

Method S15. Investigate the depletion of Cir-reporter in Auto-CAR2 system

The final concentration of Cir-reporter was fixed to 200 nM, while the concentration of Cas12a RNP ranges from 20 nM to 100 nM. Afterwards, 1 pM of target ssDNA was applied to trigger the CRISPR/Cas autocatalysis biosensing reaction. A SpectraMax iD5 multi-Mode Microplate Reader (Molecular Devices) was applied for the detection of fluorescence readout. The *E*x/*E*m of Texas red-TTATT-BHQ2 reporter was 570/615 nm.

In brief, the Auto-CAR2 reaction mixture was prepared as follows: 1 μL 100 μM (100 pmol) of Cas12a protein was gently mixed with 5 μL 20 μM (100 pmol) of gRNA-D to form the Cas12a RNP in 5 mL 1X NEB 2.1 buffer. Subsequently, 200 μL of 5 μM (1 nmol) of Cir-reporter solution was added and well mixed to form the reaction mixture, in which the final concentration of Cas12a RNP was 20 nM and Cir-reporter was 200 nM. Higher Cas12a RNP concentration was prepared by increase both the concentration of Cas12a protein and gRNA-D accordingly.

Method S16. **Evaluation and biosensing application of two Cas12a RNP based autocatalysis biosensing system (AutoCAR-3)**

The autocatalysis reaction mixture based on two RNPs was prepared as follows: 1 μL 100 μM (100 pmol) of Cas12a protein was gently mixed with 5 μL 20 μM (100 pmol) of gRNA-C to form the Cas12a RNP-1. In the meanwhile, 1 μL 100 μM (100 pmol) of Cas12a protein was gently mixed with 5 μL 20 μM (100 pmol) of gRNA-D to form the Cas12a RNP-2. Afterwards, the prepared Cas12a RNP-1 and Cas12a RNP-2 were mixed with 200 μL of 5 μM (1 nmol) of Cir-reporters in 5 mL 1X NEB 2.1 buffer to form the standard reaction mixture.

Afterwards, 10 μL of target-C ssDNA at different concentrations were added to 90 μL of the prepared reaction mixture for activating *trans*-cleavage of Cas12a and enabling the CRISPR/Cas biosensing reaction. A SpectraMax iD5 multi-Mode Microplate Reader (Molecular Devices) was applied for the detection of fluorescence readout.

Method S17. Detection of ctDNA from saliva sample

Saliva Ethics: UNSW HC200568. n=40 saliva samples were collected and stored in -20 C for further processing. The saliva samples were freeze-thawed at room temperature, then centrifuged at 2400 × g for 20 min. The supernatant was used for detection.

The AutoCAR-2 reaction mixture for ctDNA (PIK3CA H1047R) detection was prepared as follows: 1 µL 100 µM (100 pmol) of Cas12a protein was gently mixed with 5 µL 20 µM (100 pmol) of gRNA-ct to form the Cas12a RNP-1. In the meanwhile, 1 µL 100 µM (100 pmol) of Cas12a protein was gently mixed with 5 µL 20 µM (100 pmol) of gRNA-D to form the Cas12a RNP-2. Afterwards, the prepared Cas12a RNP-1 and Cas12a RNP-2 were mixed with 200 µL of 5 µM (1 nmol) of Cir-reporters in 5 mL 1X NEB 2.1 buffer to form the standard reaction mixture. Subsequently, 200 µL of 5 µM (1 nmol) of Cir-reporter solution was added and well mixed to form the reaction mixture. Afterwards, 10 µL of collected saliva sample was added to 90 µL of the prepared reaction mixture for activating *trans*-cleavage of Cas12a and enabling the CRISPR/Cas biosensing reaction. A SpectraMax iD5 multi-Mode Microplate Reader (Molecular Devices) was applied for the detection of fluorescence readout. The *E*x/*E*m of Tex-Cir-reporter-BHQ2 was 570/615 nm. All the DNA and RNA oligos used in this experiment are listed in Supplementary Table 8.

Method S18. Stability of Circular DNA structure

2 µM of Cir-reporter was prepared as descripted in Method 13 & 14. Afterwards, 90 µl of respective samples, including human plasma, saliva, and PBS buffer were mixed with 10 µl of the prepared 2 µM Cir-reporter in triplicates to the wells of 96-well plate (final concentration of Cir-reporter is 200 nM). The plate then was placed at room temperature for incubation. The degradation of Circular DNA structure then was reflected by the change of fluorescent signal, which measured at wavelengths of *E*x=570 nm and *E*m=615 nm by SpectraMax iD5 multi-Mode Microplate Reader (Molecular Devices).

Method S19. Statistical Methods

All fluorescence measurements have been made on triplicate samples (technical replicates). Justification of adequacy of triplicate testing at low molecular concentration and low sample volumes is presented below. Error bars represent standard deviation of these readings. Statistical difference or its absence (indicated by "ns") was assessed by two-tailed t-test and stated where appropriate, with stars indicating P-values (* $P<0.05$, ** $P<0.005$, *** $P<0.001$). Our experimental procedure of measuring molecular concentrations and related quantities in triplicate is not significantly affected by sampling issues at molecular concentrations and sample volumes used in this study, despite their low values. All our measurements are taken in triplicates ($X1$, $X2$, $X3$) and then we use the average value of readings X. If X represents the experimentally obtained number of molecules (the "sample estimate" in the language of statistics), it is possible to calculate the probability that this X represents the real average number of molecules, m, in these samples with 20% accuracy. To this aim we need to calculate the probability P $(0.8X < m < 1.2X)$. This probability is the same as the probability P $(10/12\, m < X < 10/8\, m) = P$ $(10/4\, m < X1+X2+X3 < 30/8\, m)$.
The random variable $X1+X2+X3$ has a Poissonian distribution with the constant $\lambda = 3m$.

Therefore $P = \sum_{\frac{10m}{4}}^{\frac{30m}{8}} \frac{(3m)^k}{k!} e^{-3m}$, and this expression makes it possible to calculate the numerical value of probability P.

For 1 aM samples at 10 µL volume used in this work for plasma measurements the value of m=10, P=0.75. For 10 aM samples at 10 µL volume, the value of m=100, and P=0.998. For larger volumes and higher concentrations P is higher than 0.998. All these probabilities are close to unity which means that our sample estimate X (the average of $X1$, $X2$, $X3$) closely

represents the value of m.

Supplementary Note

In order to understand the kinetics of the AutoCAR-1 system discussed here, we extend and adapt the model outlined in Reference [8], broadly following their notation to facilitate comparisons. We then create approximate models and find solutions which suit specific experimental conditions.

Enzyme kinetics is described by a set of nonlinear ordinary differential equations for which there are generally no closed form solutions. A common approach to deal with this problem is model reduction where simplified model equations are proposed for which solutions can be more easily obtained and analysed. The Briggs-Haldane approximation commonly adopted in the field of enzyme kinetics achieves this model reduction by forcing certain rates to be zero which replaces some of the differential equations of the model by algebraic equations. This then allows to derive the well-established Michaelis-Menten equation, which is in close agreement with many experiments. However, the understanding of reasons for this agreement require quite refined mathematical analysis [9]. It is now established that the Briggs-Haldane approximation represents the so-called singular perturbation [9]. The solutions to the original model of enzyme kinetics converge to the solutions of the reduced model satisfying the Briggs-Haldane conditions) when the ratio of enzyme to substrate concentration tends to zero [9].

Inspired by this work, our model follows the conventional Briggs-Haldane approach and Michaelis-Menten analysis for slowly varying active enzyme and other concentrations. This model is limited to the case of molecular concentration transients that are much slower than the *trans*-cleavage rate. We obtain tractable solutions for the reduced model of AutoCAR-1 kinetics. We emphasize that these do not represent the solutions of the actual AutoCAR-1 model. However, as in the case of conventional Michaelis-Menten kinetics, these solutions are close to the AutoCAR-1 solutions and they converge to them if the concentration ratio of enzyme(s) to the substrate(s) tend to zero. Therefore, as long as these ratios are small, the solutions of the reduced model are close to the experimental observables and allow to predict their trends.

Our core model describes the time evolution of molecular concentrations $[X]$ of all relevant constituents described below and highlighted in bold. Their time-dependence $\left([X]=[X](t)\right)$ is mostly omitted for clarity. The model based on coupled non-linear differential equations assumes the presence of two types of Cas12 RNPs, RNP1 and RNP2, with two different guides. It describes the evolution of targets and Cir-mediators in their two forms: original circular form and "linearized" form. References to Cir-mediators without any descriptor imply that they are in a circular form. RNP1 is bound to gRNA which matches the target sequence, while RNP2 is bound to gRNA matching the Cir-mediator dsDNA sequence. The binding of RNP1 to a target or the binding of RNP2 to a linearized Cir-mediator changes the RNP from "inactive" to "activated", a condition where the RNP becomes capable of *trans*-cleavage. Such activated RNPs cleave Cir-mediators transforming them to a linearized form. Activated RNPs are also able to cleave another molecular species we supply to the system called reporters. These reporters, like Cir-mediators have two states: they can be either uncleaved or cleaved by activated RNPs (both by RNP1 and by RNP2). The quantity observed in the experiment is the amount or the rate of increase of cleaved reporters, so our formalism is aimed at deriving and revealing the time evolution of cleaved reporter rate.

Our notation takes into consideration different roles the enzyme and substrate molecules and the reaction intermediates play in catalysis, so each molecular species will have different

symbols as its role in the catalysis process changes. Correspondingly, our kinetic equations are grouped according to the stage of the catalysis process. These stages are: (1) activation of each of the two RNPs, (2) catalytic interaction of RNPs, separately with two different substrates, (3) formation of the reaction intermediates, separately for each substrate, and (4) cleavage of each of the two substrates, one of which produces linearized Cir-mediators capable of RNP2 activation.

We denote:

$[U]$ - concentration of inactive Cas RNP1,

$[U_{cir}]$ - concentration of inactive Cas RNP2,

$[E_1]$ - concentration of activated RNP1 which are not complexed with reaction intermediates,

$[E_{1T}]$ - concentration of activated RNP1,

$[E_2]$ - concentration of activated RNP2 which are not complexed with reaction intermediates,

$[E_{2T}]$ - concentration of activated RNP2,

$[E]$ - total concentration of all activated RNPs which are not complexed with reaction intermediates,

$[E_T]$ – total concentration of activated RNP1 and RNP2,

$[T]$ - concentration of targets,

$[S_{cir}]$ - concentration of Cir-mediators in their circular form (the symbol is "S_{cir}" because they are a catalytic substrate),

$[S]$ - concentration of uncleaved reporters (the symbol is "S" because they are a catalytic substrate),

$[P_{cir}]$ - concentration of linearized (cleaved) Cir-mediators (the symbol is "P_{cir}" because they are a catalytic product),

$[P]$ - concentration of cleaved reporters (the symbol is "P" because they are a catalytic product),

$[C_{cir}]$ - concentration of reaction intermediate 1 – activated RNP (RNP1 or RNP2) and Cir-mediator complex (Cir-mediator in their circular, uncleaved form),

$[C]$ - concentration of reaction intermediate 2 – activated RNP (RNP1 or RNP2) and uncleaved reporter complex.

The species conservation rules are as follows:

$[E_{1T}]+ [E_{2T}]=[E_T]$ (activated RNP1 + activated RNP2 = total activated RNPs).

$[E_T] = [E] + [C] + [C_{cir}]$ (total activated RNPs = total activated uncomplexed RNPs+ concentration of both complexes)

$T_0 - [T] = [E_{1T}]$ (initial targets – unbound targets = activated RNP1). Similar simple condition can not be written for $[E_{2T}]$ because Cir-mediators do not only activate RNP2 but also take part in catalysis.

$[C](t) + [S](t)+[P](t)=S_0$ (reporters complexed with RNPs + uncleaved reporters+ cleaved reporters = initial concentration of reporters)

$[C_{cir}](t)$+$[S_{cir}](t)$+$[P_{cir}](t)$=S_{cir0} (Cir-mediators complexed with RNPs + uncleaved Cir-mediators (in their circular form) + cleaved (linearized) Cir-mediators = initial concentration of supplied Cir-mediators in their circular form)

We note here that unlike in Ref. 1 the species conservation condition for activated RNPs is invalid in our problem, as these concentrations are varying in autocatalysis.

The initial conditions are:

$[E_{1T}](0)$= $[E_{2T}](0)$=0 - initial concentrations of activated RNPs expressing no initial RNP activation,

U_0 - initial concentration of RNP1,

U_{cir0} - initial concentration of RNP2,

S_{cir0}- initial concentration of Cir-mediators in their circular form,

S_0 - initial concentration of uncleaved reporters,

P_{cir0} = 0 - initial concentration of linearized (cleaved) Cir-mediators,

P_0 = 0 - initial concentration of cleaved reporters.

We also denote:

C_0 – a constant describing the concentration of the reaction intermediate for RNPs with uncleaved reporters according to the Briggs-Haldane approximation.

C_{cir0} - a constant describing the concentration of the reaction intermediate for RNPs with uncleaved Cir-mediators according to the Briggs-Haldane approximation.

Other constants, especially those resulting from integration and symbols used to facilitate algebra are defined in the text.

The reactions describing the activation of the two RNPs can be modelled as:

$U+T \leftrightarrow E_{1T}$ with bimolecular association and dissociation rate constants k_{on}, k_{off}

$U_{cir}+P_{cir} \leftrightarrow E_{2T}$ with are bimolecular association and dissociation rate constants k_{on}, k_{off}

The rate equations for RNP activation are:

$$\frac{d[E_{1T}]}{dt} = k_{on}[U][T]- k_{off}[E_{1T}] \qquad /1/$$

$$\frac{d[E_{2T}]}{dt} = k_{on}[U_{cir}][P_{cir}] - k_{off}[E_{2T}] \qquad /2/$$

where the first term on the right describes the rate of RNP-target complex formation (which leads to activation, hence + sign) and the second term describes the decomposition of the complex (and deactivation). We assume here that the on and off reaction rates are identical for targets and Cir-mediators.

The catalytic reactions are modelled following the approach in Ref 1. The catalytic reaction of reporter cleavage is modelled as:

$$E+S \overset{(k_f,k_r)}{\leftrightarrow} C \overset{(k_{cat})}{\rightarrow} E+P \qquad /3/$$

The catalytic reaction of Cir-mediator cleavage (linearisation) is modelled as:

$$(k_f,k_r) \qquad (k_{cat})$$

$$E+S_{\mathrm{cir}} \quad \leftrightarrow \quad C_{\mathrm{cir}} \quad \rightarrow E+P_{\mathrm{cir}} \qquad /4/$$

Here, (k_f,k_r) are bimolecular association and dissociation rate constants for the reaction between activated RNP, uncleaved reporters and Cir-mediators in circular form, while k_{cat} is the cleavage rate of reporters and Cir-mediators. As a first approximation, we assume that these rates are the same for reporters and Cir-mediators although the nucleic acid sequences differ. (The literature reports these rates to be somewhat sequence - dependent).

The Cir-mediator system is described by the following system of nonlinear differentialequations:

The "catalysis" equations:

$$\frac{d[E]}{dt} = - k_f[E][S] - k_f[E][S_{\mathrm{cir}}] + k_r[C] + k_r[C_{\mathrm{cir}}] + k_{\mathrm{cat}}[C] + k_{\mathrm{cat}}[C_{\mathrm{cir}}] \qquad /5/$$

where

$$[E]=[E_1]+[E_2] \qquad /6/$$

In Equation /5/ the first term on the right has a minus sign because the formation of the RNP-substrate complex decreases the concentration of RNP available for further complex formation.

The "substrate" equations are, correspondingly:

$$\frac{d[S]}{dt} = - k_f[E][S] + k_r[C] \qquad /7/$$

$$\frac{d[S_{\mathrm{cir}}]}{dt} = - k_f[E][S_{\mathrm{cir}}] + k_r[C_{\mathrm{cir}}] \qquad /8/$$

The equations describing how the reaction intermediates are formed are given by:

$$\frac{d[C]}{dt} = k_f[E][S] - k_r[C] - k_{\mathrm{cat}}[C] \qquad /9/$$

$$\frac{d[C_{\mathrm{cir}}]}{dt} = k_f[E][S_{\mathrm{cir}}] - k_r[C_{\mathrm{cir}}] - k_{\mathrm{cat}}[C_{\mathrm{cir}}] \qquad /10/$$

The "product" equations are:

$$\frac{d[P]}{dt} = k_{\mathrm{cat}}[C] \qquad /11/$$

$$\frac{d[P_{\mathrm{cir}}]}{dt} = k_{\mathrm{cat}}[C_{\mathrm{cir}}] \qquad /12/$$

These equations, together with species conservation conditions and initial conditions, allow to predict all relevant concentrations in this problem.

We now analyse the results derived from these equations from a number of important perspectives, and in practically relevant cases. As usual in catalysis, the ratios of substrates to the relevant enzymes cleaving those substrates are particularly significant, but in this specific case of catalysis mediated by Cir-mediators, other ratios including the ratio of targets to Cir-mediators are also important. In the below, in all sections of our analysis, we will generally assume that the concentrations of reporters is much higher than the initial concentration of both RNPs. We will also assume that initial concentrations of both RNPs are high compared with the concentration of the respective activated RNPs. In some instances, the time-dependence of these concentrations may be ignored and the concentrations replaced by the initial values ($[S]=S_0$, $[U]=U_0$, $[U_{\mathrm{cir}}]=U_{\mathrm{cir0}}$).

In the below the cleaved reporter rate $\frac{d[P]}{dt}$ is referred to as the "**experimental observable**".

Result 1: The experimental observable reflects the concentration of activated RNPs in the Briggs-Haldane approximation.

Derivation:

We apply the Briggs-Haldane approximation to the reporters and to Cir-mediators. This approximation says that the concentrations of reaction intermediates are constant ($[C]$ = const, $[C_{\text{cir}}]$ = const). We should emphasise here that the Briggs-Haldane approximation is strictly not applicable to the very initiation of catalysis because the concentration of reaction intermediates (in this case $[C]$) at t=0 is zero.

We equate $\frac{d([C])}{dt}$ and $\frac{d([C_{\text{cir}}])}{dt}$ to zero in Equations /9/ and /10/ and add these two equations to get:

$$\frac{d([C]+[C_{\text{cir}}])}{dt} = 0 = k_{\text{f}}[E]([S]+[S_{\text{cir}}]) - (k_{\text{r}} + k_{\text{cat}})([C]+[C_{\text{cir}}]) \qquad /13/$$

By adding Equation /11/ and Equation /12/ we get the expression for the rate of product formation for the reporters and for Cir-mediators, the first of which contains the experimental observable.

$$\frac{d([P]+[P_{\text{cir}}])}{dt} = k_{\text{cat}}([C]+[C_{\text{cir}}]) \qquad /14/$$

Equation /13/ leads to the familiar form of the Michaelis-Menten equation, in the following way:

$$k_{\text{f}}[E]([S]+[S_{\text{cir}}]) = (k_{\text{r}} + k_{\text{cat}})([C]+[C_{\text{cir}}]) \qquad /15/$$

We remind that

$$[E_T] = [E]+[C]+[C_{\text{cir}}] \qquad /16/$$

so we can now substitute

$$[E] = [E_{\text{T}}] - ([C]+[C_{\text{cir}}]) \qquad /17/$$

producing

$$k_{\text{f}}([E_{\text{T}}] - ([C]+[C_{\text{cir}}])([S]+[S_{\text{cir}}]) = (k_{\text{r}} + k_{\text{cat}})([C]+[C_{\text{cir}}]) \qquad /18/$$

So

$$k_{\text{f}}[E_{\text{T}}]([S]+[S_{\text{cir}}]) - k_{\text{f}}([C]+[C_{\text{cir}}])([S]+[S_{\text{cir}}]) = (k_{\text{r}} + k_{\text{cat}})([C]+[C_{\text{cir}}]) \qquad /19/$$

And

$$k_{\text{f}}[E_{\text{T}}]([S]+[S_{\text{cir}}]) = (k_{\text{r}} + k_{\text{cat}})([C]+[C_{\text{cir}}]) + k_{\text{f}}([C]+[C_{\text{cir}}])([S]+[S_{\text{cir}}]) \qquad /20/$$

which means that

$$[C]+[C_{\text{cir}}] = \frac{k_{\text{f}}[E_{\text{T}}]([S]+[S_{\text{cir}}])}{k_{\text{r}} + k_{\text{cat}} + k_{\text{f}}([S]+[S_{\text{cir}}])} \qquad /21/$$

Hence from Equation /14/ and Equation/21/ the rate $\frac{d([P]+[P_{\text{cir}}])}{dt}$ can be expressed as:

$$\frac{d([P]+[P_{\text{cir}}])}{dt} = k_{\text{cat}}([C]+[C_{\text{cir}}]) = \frac{k_{\text{cat}} k_{\text{f}}[E_{\text{T}}]([S]+[S_{\text{cir}}])}{k_{\text{r}} + k_{\text{cat}} + k_{\text{f}}([S]+[S_{\text{cir}}])} \qquad /22/$$

which can be expressed using the Michaelis-Menten constant $K_{\text{M}} = \frac{(k_{\text{r}} + k_{\text{cat}})}{k_{\text{f}}}$ as

$$\frac{d([P]+[P_{\text{cir}}])}{dt} = \frac{k_{\text{cat}}[E_{\text{T}}]([S]+[S_{\text{cir}}])}{K_{\text{M}} + ([S]+[S_{\text{cir}}])} \qquad /23/$$

In conditions when $\frac{d[P_{\text{cir}}]}{dt} \ll \frac{d[P]}{dt}$, for example when the concentration of Cir-mediators is much lower than the concentration of reporters, or Cir-mediators are close to being depleted and $\frac{d[P_{\text{cir}}]}{dt}$ is approximately zero, we get the following approximate expression for the experimental observable:

$$\frac{d[P]}{dt} = \frac{k_{\text{cat}}[E_{\text{T}}]([S]+[S_{\text{cir}}])}{K_{\text{M}}+([S]+[S_{\text{cir}}])} \qquad /24/$$

This means that the experimental observable follows the time-varying concentration of active RNPs, in analogy with standard CRISPR/Cas reaction without autocatalysis. Furthermore, in conditions when the Cir-mediators are close to being depleted, the reaction kinetics returns to a linear trend with reaction time, as in a standard CRISPR/Cas reaction system and the experimental observable is then proportional to the total concentration of RNPs successfully activated by targets and linearized Cir-mediators.

Result 2: When the RNP2 and Cir-mediators are sufficiently abundant, much more abundant than targets, and if RNP2 and Cir-mediators do not get noticeably depleted, then the experimental observable increases approximately exponentially.

Derivation:

Cir-mediators sufficiently abundant means that $[S_{\text{cir}}]=S_{\text{cir0}}$, and we are also assuming that $S_{\text{cir0}} \gg [T]$. Sufficiently abundant RNP2 means that $[U_{\text{cir}}]$ can be approximated by U_{cir0}.

The relevant activation equation in our approximation where we have many more Cir-mediators than targets is Equation /2/ where we now replace $[E_{2\text{T}}]$ by $[E_{\text{T}}]$.

$$\frac{d[E_{\text{T}}]}{dt} = k_{\text{on}}U_{\text{cir0}}[P_{\text{cir}}] - k_{\text{off}}[E_{\text{T}}] \qquad /25/$$

We now apply the Briggs -Haldane approximation to the Cir-mediators in Equation /10/. We obtain:

$$\frac{d[C_{\text{cir}}]}{dt} = 0 = k_{\text{f}}[E][S_{\text{cir}}] - k_{\text{r}}[C_{\text{cir}}] - k_{\text{cat}}[C_{\text{cir}}] \qquad /26/$$

Hence

$$[C_{\text{cir}}] = k_{\text{f}}[E][S_{\text{cir}}] \big/ (k_{\text{r}}+k_{\text{cat}}) \qquad /27/$$

The above Equation /27/ and Equation/12/ lead to a simple expression for the rate of product formation for the reporters (which represent the experimental observable).

$$\frac{d[P_{\text{cir}}]}{dt} = k_{\text{cat}}[C_{\text{cir}}] = k_{\text{cat}}k_{\text{f}}[E][S_{\text{cir}}] \big/ (k_{\text{r}}+k_{\text{cat}}) \qquad /28/$$

In our special case

$$\frac{d[P_{\text{cir}}]}{dt} = k_{\text{cat}}[C_{\text{cir}}] = k_{\text{cat}}k_{\text{f}}[E]\, S_{\text{cir0}} \big/ (k_{\text{r}}+k_{\text{cat}}) \qquad /29/$$

We now substitute one of the species conservation rules, which in Briggs-Haldane approximation says that $[E_{\text{T}}]=[E]+(C_0+C_{\text{cir0}})$. From Equation /28/ we get:

$$\frac{d[P_{\text{cir}}]}{dt} = k_{\text{cat}}k_{\text{f}}[E]S_{\text{cir0}} \big/ (k_{\text{r}}+k_{\text{cat}}) = \frac{k_{\text{cat}}k_{\text{f}}\, S_{\text{cir0}}}{(k_{\text{r}}+k_{\text{cat}})} *([E_{\text{T}}]-(C_0+C_{\text{cir0}})) \qquad /30/$$

For brevity, we denote $C_0+C_{\text{cir0}}=C_1$. Formally Equations /25/ and /30/ have the form:

$$\frac{d[E_{\text{T}}]}{dt} = p[P_{\text{cir}}] - k_{\text{off}}[E_{\text{T}}] \qquad /31/$$

$$\frac{d[P_{\text{cir}}]}{dt} = q([E_T] - C_1) \qquad /32/$$

where $p = k_{\text{on}}\ U_{\text{cir0}}$ and $q = \left. k_{\text{cat}} k_f S_{\text{cir0}} \middle/ (k_r + k_{\text{cat}}) \right.$.

From Equations /31/ and /32/ we get:

$$\frac{d^2[E_T]}{dt^2} + k_{\text{off}} \frac{d[E_T]}{dt} - pq[E_T] + pqC_1 = 0 \qquad /33/$$

The solution is a sum of a general solution of a homogeneous equation and a specific solution of an inhomogeneous equation. We can guess that specific solution by noticing that a constant E_s can satisfy the inhomogeneous equation as long as:

$$-pqE_s + pqC_1 = 0 \qquad /34/$$

so

$$E_s = C_1 \qquad /35/$$

Consequently,

$$[E_T] = A\ exp(\lambda_1\ t) + B\ \ exp(\lambda_2\ t) + C_1 \qquad /36/$$

where $\lambda_1 = \frac{-k_{\text{off}} + \sqrt{k_{\text{off}}^2 + 4pq}}{2}$ and $\lambda_2 = \frac{-k_{\text{off}} - \sqrt{k_{\text{off}}^2 + 4pq}}{2}$ and A, B are constants obtained from the initial conditions ($[E_T](0) = 0$, and $\frac{d[E_T]}{dt}(0) = 0$).

In this solution, the first exponentially increasing term dominates at increasing times.

The total cleavage rates of linearised Cir-mediators and reporters are proportional to their concentrations. Therefore, if the concentration of linearized Cir-mediators is much lower than the concentration of cleaved reporters, Equation /24/ leads to a following approximate expression for the experimental observable:

$$\frac{d[P]}{dt} = \frac{k_{\text{cat}}[E_T]([S]+[S_{\text{cir}}])}{K_M + ([S]+[S_{\text{cir}}])} = \frac{k_{\text{cat}}([S]+[S_{\text{cir}}])}{(K_M + ([S]+[S_{\text{cir}}]))} * \left(A\ exp(\lambda_1\ t) + B\ \ exp(\lambda_2\ t) + C_1\right) \qquad /37/$$

Hence at increasing times under the above experimental conditions we should observe the fluorescence rates increasing approximately exponentially. The fluorescence signal is proportional to the integral of fluorescence rate. Exponentially increasing rate produces a signal exponentially increasing with time.

Adaptation of the AutoCAR-1 model to the case of the AutoCAR-2 system.

We denote:

U_0 - initial concentration of supplied RNPs,

$[U]$- concentration of inactive Cas RNPs

$[E]$ - total concentration of activated RNPs which are not complexed with reaction intermediates,

$[E_T]$ – total concentration of activated RNPs,

$[T]$ - concentration of targets,

$[S_{\text{cir}}]$ - concentration of Cir-reporters in their circular form

$[P_{cir}]$ - concentration of linearised (cleaved) Cir-reporters

$[C_{cir}]$ - concentration of reaction intermediates – activated RNP and Cir-reporter complexes (Cir-reporters in their circular, uncleaved form),

The species conservation conditions and their meaning are as follows:

$[E_T]=[E]+[C_{cir}]$ concentration of total activated RNPs = concentration of total activated uncomplexed RNPs + concentration of the reaction intermediates).

$U_0=[U]+[E_T]$ initial concentration of RNPs = sum of inactive and active RNPs

$[C_{cir}]+[S_{cir}]+[P_{cir}]=S_{cir0}$ Cir-reporters complexed with RNPs + uncleaved Cir-reporters in their circular form + cleaved (linearised) Cir-reporters = initial concentration of supplied Cir-reporters in their circular form.

The initial conditions are:

$[E_T](0) = 0$ - initial concentrations of activated RNPs expressing no initial RNP activation,

U_0 - initial concentration of RNPs,

S_{cir0}- initial concentration of Cir-reporters in their circular form,

$P_{cir0} = 0$ - initial concentration of linearised (cleaved) Cir-reporters,

We also denote:

C_{cir0} - a constant describing the concentration of the reaction intermediate for RNPs with uncleaved Cir-reporters according to the Briggs-Haldane approximation.

The reactions describing the activation of the RNPs can be modelled as:

$U+T+P_{cir} \leftrightarrow E_T$ with bimolecular association and dissociation rate constants k_{on}, k_{off}

The rate equation for RNP activation is:

$$\frac{d[E_T]}{dt} = k_{on}[U]([T]+[P_{cir}]) - k_{off}[E_T] \qquad /38/$$

The catalytic reaction of Cir-reporter cleavage (linearisation) is modelled as:

$$E+S_{cir} \overset{(k_f,\, k_r)}{\leftrightarrow} C_{cir} \overset{(k_{cat})}{\rightarrow} E+P_{cir} \qquad /39/$$

Here, (k_f, k_r) are bimolecular association and dissociation rate constants for the reaction between activated RNP and Cir-reporters in circular form, while k_{cat} is the cleavage rate of Cir-reporter.

The Cir-reporter system is described by the following system of nonlinear differential equations:

The “catalysis” equation is:

$$\frac{d[E]}{dt} = - k_f[E][S_{cir}]+(k_r+k_{cat})[C_{cir}] \qquad /40/$$

The “substrate” equation is, correspondingly:

$\frac{d[S_{cir}]}{dt} = - k_f[E][S_{cir}] + k_r[C_{cir}]$ /41/

The "intermediate" equation describing how the reaction intermediates are formed is given by:

$\frac{d[C_{cir}]}{dt} = k_f[E][S_{cir}] - (k_r + k_{cat})[C_{cir}]$ /42/

The "product" equation is:

$\frac{d[P_{cir}]}{dt} = k_{cat}[C_{cir}]$ /43/

These equations, together with species conservation conditions and initial conditions, allow to predict all relevant concentrations in this problem.

In the below the cleaved Cir-reporter rate $\frac{d[P_{cir}]}{dt}$ is referred to as the "experimental observable".

Result 3: The experimental observable reflects the concentration of activated RNPs in the Briggs-Haldane approximation.

Derivation:

We first apply the standard approximation (Briggs-Haldane) to the Cir-reporter. This approximation says that the concentration of reaction intermediates is constant ($[C_{cir}]$ = const)

From Equation /10/ we then get

$$\frac{d[C_{cir}]}{dt} = 0 = k_f[E][S_{cir}] - (k_r + k_{cat})[C_{cir}]$$

Hence

$$k_f[E][S_{cir}] = (k_r + k_{cat})[C_{cir}]$$

This leads to the Michaelis-Menten equation, in the following way. We substitute in the above equation

$$[E] = [E_T] - [C_{cir}]$$

$$k_f([E_T] - [C_{cir}])[S_{cir}] = (k_r + k_{cat})[C_{cir}]$$

This gives

$$[C_{cir}] = \frac{k_f[E_T]([S_{cir}])}{k_r + k_{cat} + k_f([S_{cir}])}$$

Hence from Equation /16/ the rate $\frac{d([P_{cir}])}{dt}$ can be expressed as:

$$\frac{d([P_{cir}])}{dt} = k_{cat}([C_{cir}]) = \frac{k_{cat}k_f[E_T]([S_{cir}])}{k_r + k_{cat} + k_f([S_{cir}])}$$

which can be expressed using the Michaelis-Menten constant $K_M = \frac{(k_r + k_{cat})}{k_f}$ as

$$\frac{d([P_{cir}])}{dt} = \frac{k_{cat}[E_T][S_{cir}]}{K_M + [S_{cir}]}$$

which is very similar to a standard Michaelis-Menten equation except that $[E_T]$ varies over the course of the reaction.

Result 4: When the RNPs and Cir-reporters are sufficiently abundant and do not get depleted, the experimental observable increase approximately exponentially.

Derivation: The condition that Cir-reporters are sufficiently abundant means that $[S_{cir}] = S_{cir0}$, and we are also assuming here that $S_{cir0} \gg [T]$. Sufficiently abundant RNPs means that $[U_{cir}]$ can be approximated by U_{cir0}.We will assume that S_{cir0} and $[E]$ are comparable and that the Briggs-Haldane approximation is also applicable to Cir-reporter, so $[C_{cir}]$ = const.

$$\frac{d([P_{cir}])}{dt} = \frac{k_{cat}[E_T][S_{cir}]}{K_M + [S_{cir}]} = \frac{k_{cat}[E_T]S_{cir0}}{K_M + S_{cir0}}$$

Hence, in order to evaluate the experimental observable, we need to evaluate $[E_T]$.

The relevant activation equation in our approximation where we have many more Cir-reporters than targets is derived from Equation /1/ as:

$$\frac{d[E_T]}{dt} = k_{on}U_{cir0}[P_{cir}] - k_{off}[E_T] \qquad /44/$$

Combining Equation /12/ with Equation /10/ where $\frac{d[C_{cir}]}{dt} = 0$ we get in our regime and after the application of Briggs-Haldane approximation:

$$\frac{d[P_{cir}]}{dt} = {k_{cat}k_f[E]S_{cir0}}\Big/{(k_r + k_{cat})} = \frac{k_{cat}k_f S_{cir0}}{(k_r + k_{cat})}([E_T] - C_{cir0}) \qquad /45/$$

Equations /19/ and /20/ have the form:

$$\frac{d[E_T]}{dt} = p[P_{cir}] - k_{off}[E_T] \qquad /46/$$

$$\frac{d[P_{cir}]}{dt} = q([E_T] - C_{cir0}) \qquad /47/$$

where $p = k_{on}\,U_{cir0}$ and

$$q = {k_{cat}k_f S_{cir0}}\Big/{(k_r + k_{cat})}$$

From Equations /21/ and /22/ we get:

$$\frac{d^2[E_T]}{dt^2} + k_{off}\frac{d[E_T]}{dt} - pq[E_T] + pqC_{cir0} = 0 \qquad /48/$$

The solution of Equation /23/ is a sum of a general solution of a homogeneous equation and a specific solution of an inhomogeneous equation. We can guess that specific solution by noticing that a constant E_s can satisfy the inhomogeneous equation as long as:

$$-pqE_s + pqC_{cir0} = 0$$

so

$$E_s = C_{cir0}$$

Consequently,

$$[E_T] = A\,exp(\lambda_1\,t) + B\,\,exp(\lambda_2\,t) + C_{cir0} \qquad /49/$$

where $\lambda_1 = \frac{-k_{off} + \sqrt{k_{off}^2 + 4pq}}{2}$ and $\lambda_2 = \frac{-k_{off} - \sqrt{k_{off}^2 + 4pq}}{2}$ and A, B are constants obtained from the initial conditions ($[E_T](0) = 0$, and $\frac{d[E_T]}{dt}(0) = 0$).

We can use Equation /18/ to express the experimental observable:

$$\frac{d[P_{cir}]}{dt} = \frac{k_{cat}[E_T][S_{cir}]}{K_M + [S_{cir}]} =$$

$$= \frac{k_{cat}[S_{cir}]}{K_M + [S_{cir}]}(A\,exp(\lambda_1\, t) + B\ exp(\lambda_2\, t) + C_{cir0}) \qquad /50/$$

Hence at increasing times under the above experimental conditions we should observe the fluorescence rates in AutoCAR-2 increasing approximately exponentially. The fluorescence signal is proportional to the integral of fluorescence rate. Exponentially increasing rate produces a signal exponentially increasing with time.

At increasing times, the solution is dominated by the term $exp(\lambda_1\, t)$ .The exponent λ_1 can be experimentally controlled via pq, which is a product of various molecular constants and $S_{cir0} U_{cir0}$. Correspondingly, to maximise the reaction rate in the reaction mixture, the concentration of enzyme and substrate should be maximised, while making sure that the substrate is not depleted.

References

1. Zuker, M. Mfold web server for nucleic acid folding and hybridization prediction. *Nucleic Acids Res* **31**, 3406-3415 (2003).
2. Turner, D.H. & Mathews, D.H. NNDB: the nearest neighbor parameter database for predicting stability of nucleic acid secondary structure. *Nucleic Acids Res* **38**, D280-D282 (2010).
3. An, R. et al. Highly efficient preparation of single-stranded DNA rings by T4 ligase at abnormally low Mg(II) concentration. *Nucleic Acids Res* **45** (2017).
4. Chao, J. et al. Click-chemistry-conjugated oligo-angiomax in the two-dimensional DNA lattice and its interaction with thrombin. *Biomacromolecules* **10**, 877-883 (2009).
5. Taemaitree, L., Shivalingam, A., El-Sagheer, A.H. & Brown, T. An artificial triazole backbone linkage provides a split-and-click strategy to bioactive chemically modified CRISPR sgRNA. *Nat Commun* **10**, 1610 (2019).
6. Holmberg, A. et al. The biotin-streptavidin interaction can be reversibly broken using water at elevated temperatures. *Electrophoresis* **26**, 501-510 (2005).
7. Chen, J., Baker, Y.R., Brown, A., El-Sagheer, A.H. & Brown, T. Enzyme-free synthesis of cyclic single-stranded DNA constructs containing a single triazole, amide or phosphoramidate backbone linkage and their use as templates for rolling circle amplification and nanoflower formation. *Chemical science* **9**, 8110-8120 (2018).
8. Ramachandran, A. & Santiago, J.G. CRISPR Enzyme Kinetics for Molecular Diagnostics. *Anal Chem* **93**, 7456-7464 (2021).
9. Heineken, F.G., Tsuchiya, H.M. & Aris, R. On the mathematical status of the pseudo-steady state hypothesis of biochemical kinetics. *Mathematical Biosciences* **1**, 95-113 (1967).
