# Supplementary material for: Topological barrier to Cas12a activation by circular DNA nanostructures facilitates autocatalysis and transforms DNA/RNA sensing
Source: Nat Commun. 2024 Mar 5;15:1818. doi: 10.1038/s41467-024-46001-8 (PMC10914725; doi:10.1038/s41467-024-46001-8)
Supplement: Supplementary file 2 — Reporting Summary [file 41467_2024_46001_MOESM2_ESM.pdf]

nature portfolio

Corresponding author(s): Yi Li

Last updated by author(s): Jan 19, 2024

Reporting Summary

Nature Portfolio wishes to improve the reproducibility of the work that we publish. This form provides structure for consistency and transparency in reporting. For further information on Nature Portfolio policies, see our Editorial Policies and the Editorial Policy Checklist.

Statistics

For all statistical analyses, confirm that the following items are present in the figure legend, table legend, main text, or Methods section.

n/a	Confirmed	
☐	☒	The exact sample size (*n*) for each experimental group/condition, given as a discrete number and unit of measurement
☐	☒	A statement on whether measurements were taken from distinct samples or whether the same sample was measured repeatedly
☐	☒	The statistical test(s) used AND whether they are one- or two-sided<br>*Only common tests should be described solely by name; describe more complex techniques in the Methods section.*
☒	☐	A description of all covariates tested
☒	☐	A description of any assumptions or corrections, such as tests of normality and adjustment for multiple comparisons
☐	☒	A full description of the statistical parameters including central tendency (e.g. means) or other basic estimates (e.g. regression coefficient) AND variation (e.g. standard deviation) or associated estimates of uncertainty (e.g. confidence intervals)
☐	☒	For null hypothesis testing, the test statistic (e.g. *F*, *t*, *r*) with confidence intervals, effect sizes, degrees of freedom and *P* value noted<br>*Give P values as exact values whenever suitable.*
☒	☐	For Bayesian analysis, information on the choice of priors and Markov chain Monte Carlo settings
☒	☐	For hierarchical and complex designs, identification of the appropriate level for tests and full reporting of outcomes
☒	☐	Estimates of effect sizes (e.g. Cohen's *d*, Pearson's *r*), indicating how they were calculated

Our web collection on statistics for biologists contains articles on many of the points above.

Software and code

Policy information about availability of computer code

Data collection	The fluorescence signal intensities in this study was determined and collected by using Spectra Max iD5 Multi-Mode Microplate Reader (Molecular Devices) . PCR data were collected using Bio-Rad CFX96 Real-Time PCR machine.
Data analysis	Office Excel v2321 and Prism 9 v9.4.1 softwares were used to generate two tail T-test statistic analysis results, and also equations for curves. ImageJ 1.53t software was used to analyze the gel image. Origin 2022 v9.9 was used to analysis the kinetic data generated by the iD5 Multi-Mode Microplate Reader.

For manuscripts utilizing custom algorithms or software that are central to the research but not yet described in published literature, software must be made available to editors and reviewers. We strongly encourage code deposition in a community repository (e.g. GitHub). See the Nature Portfolio guidelines for submitting code & software for further information.

Data

Policy information about availability of data

All manuscripts must include a data availability statement. This statement should provide the following information, where applicable:

- Accession codes, unique identifiers, or web links for publicly available datasets
- A description of any restrictions on data availability
- For clinical datasets or third party data, please ensure that the statement adheres to our policy

Source data are provided with this paper. No restriction on data availability.

PDB data used in this study including: 5XUS [https://doi.org/10.2210/pdb5XUS/pdb], 6NME [https://doi.org/10.2210/pdb6NME/pdb]

Research involving human participants, their data, or biological material

Policy information about studies with human participants or human data. See also policy information about sex, gender (identity/presentation), and sexual orientation and race, ethnicity and racism.

Reporting on sex and gender	Not relevant. as this is biochemistry and not biomedical research. Human blood samples were used for verifying the function of the developed system. The human (patient) bloods are provided by the Cancer Molecular Screening and Therapeutics (MoST) program (ACTRN12616000908437). The MoST program was performed in accordance with the Declaration of Helsinki. The program has been approved by the St Vincent's Hospital Sydney Human Research Ethics Committee (reference, HREC/16/SVH/23). Total of 10 blood samples (10 female) were used in this study, including 6 positive and 4 negative for PIK3CA H1047R mutation. All human plasma experiments were approved by the UNSW Ethics Committee (UNSW HC210160), in addition to ACTRN12616000908437. All human saliva experiments were approved by the UNSW Ethics Committee (UNSW HC200568).
Reporting on race, ethnicity, or other socially relevant groupings	Not relevant.
Population characteristics	Not relevant.
Recruitment	Not relevant.
Ethics oversight	UNSW Ethics Committee, St Vincent's Hospital Sydney Human Research Ethics Committee

Note that full information on the approval of the study protocol must also be provided in the manuscript.

Field-specific reporting

Please select the one below that is the best fit for your research. If you are not sure, read the appropriate sections before making your selection.

- [x] Life sciences
- [ ] Behavioural & social sciences
- [ ] Ecological, evolutionary & environmental sciences

For a reference copy of the document with all sections, see nature.com/documents/nr-reporting-summary-flat.pdf

Life sciences study design

All studies must disclose on these points even when the disclosure is negative.

Sample size	This is a nucleic acid diagnostic method development study, if the sample size refers to the tested reactions for each experiment, 3 independent reactions are applied for detection to generate 3 independent fluorescence signals. In diagnostic applications, for testing each sample, 3 independent reactions (tests) are the generally accepted approach.
Data exclusions	No experimental data was excluded.
Replication	For each of the test in the manuscript, 3 independent reactions are applied for detection to generate 3 independent fluorescence signals. All attempts at 3 independent reactions were successful.
Randomization	Both the tested synthetic DNA/RNA oligos or the genome DNA/RNA are randomly taken and allocated into independent reactions.
Blinding	The used DNA/RNA oligos for designing CRISPR/Cas12a biosensing system are not used randomly because the nature of biosensor design requires understanding of the components details. The genome DNA/RNA used as samples were blinded before detection using the developed biosensing system, in order to avoid bias.

Reporting for specific materials, systems and methods

We require information from authors about some types of materials, experimental systems and methods used in many studies. Here, indicate whether each material, system or method listed is relevant to your study. If you are not sure if a list item applies to your research, read the appropriate section before selecting a response.

Materials & experimental systems

n/a	Involved in the study
☐	☒ Antibodies
☐	☒ Eukaryotic cell lines
☒	☐ Palaeontology and archaeology
☐	☒ Animals and other organisms
☒	☐ Clinical data
☒	☐ Dual use research of concern
☒	☐ Plants

Methods

n/a	Involved in the study
☒	☐ ChIP-seq
☒	☐ Flow cytometry
☒	☐ MRI-based neuroimaging

Antibodies

Antibodies used	The antibody (anti-FAM antibody) is an implemented reagent on the pre-made lateral flow strip, which is commercially purchased from HybriDetect – Universal Lateral Flow Assay Kit (Millenia Biotec).
Validation	Not relevant.

Eukaryotic cell lines

Policy information about cell lines and Sex and Gender in Research

Cell line source(s)	HCT-116-Luc2, Cat. CCL-247-LUC2, ATCC
Authentication	Not relevant for this study.
Mycoplasma contamination	Not relevant for this study.
Commonly misidentified lines (See ICLAC register)	Not relevant for this study.

Animals and other research organisms

Policy information about studies involving animals; ARRIVE guidelines recommended for reporting animal research, and Sex and Gender in Research

Laboratory animals	NOD/SCID (6-8-week-old) mice
Wild animals	Not relevant.
Reporting on sex	Not relevant.
Field-collected samples	Not relevant.
Ethics oversight	UNSW Animal Care and Ethics Committee (UNSW animal research ethics project approval 20/95B, 21/39B, and 21/77B)

Note that full information on the approval of the study protocol must also be provided in the manuscript.

Plants

Seed stocks	Not relevant.
Novel plant genotypes	Not relevant.
Authentication	Not relevant.
